# Supplementary material for: Outward-oriented sites within clustered CTCF boundaries are key for intra-TAD chromatin interactions and gene regulation
Source: Nat Commun. 2023 Dec 7;14:8101. doi: 10.1038/s41467-023-43849-0 (PMC10703910; doi:10.1038/s41467-023-43849-0)
Supplement: Supplementary file 1 — Supplementary Information [file 41467_2023_43849_MOESM1_ESM.pdf]

Outward-oriented sites within clustered CTCF boundaries are key for intra-TAD chromatin interactions and gene regulation

Ge *et al.*

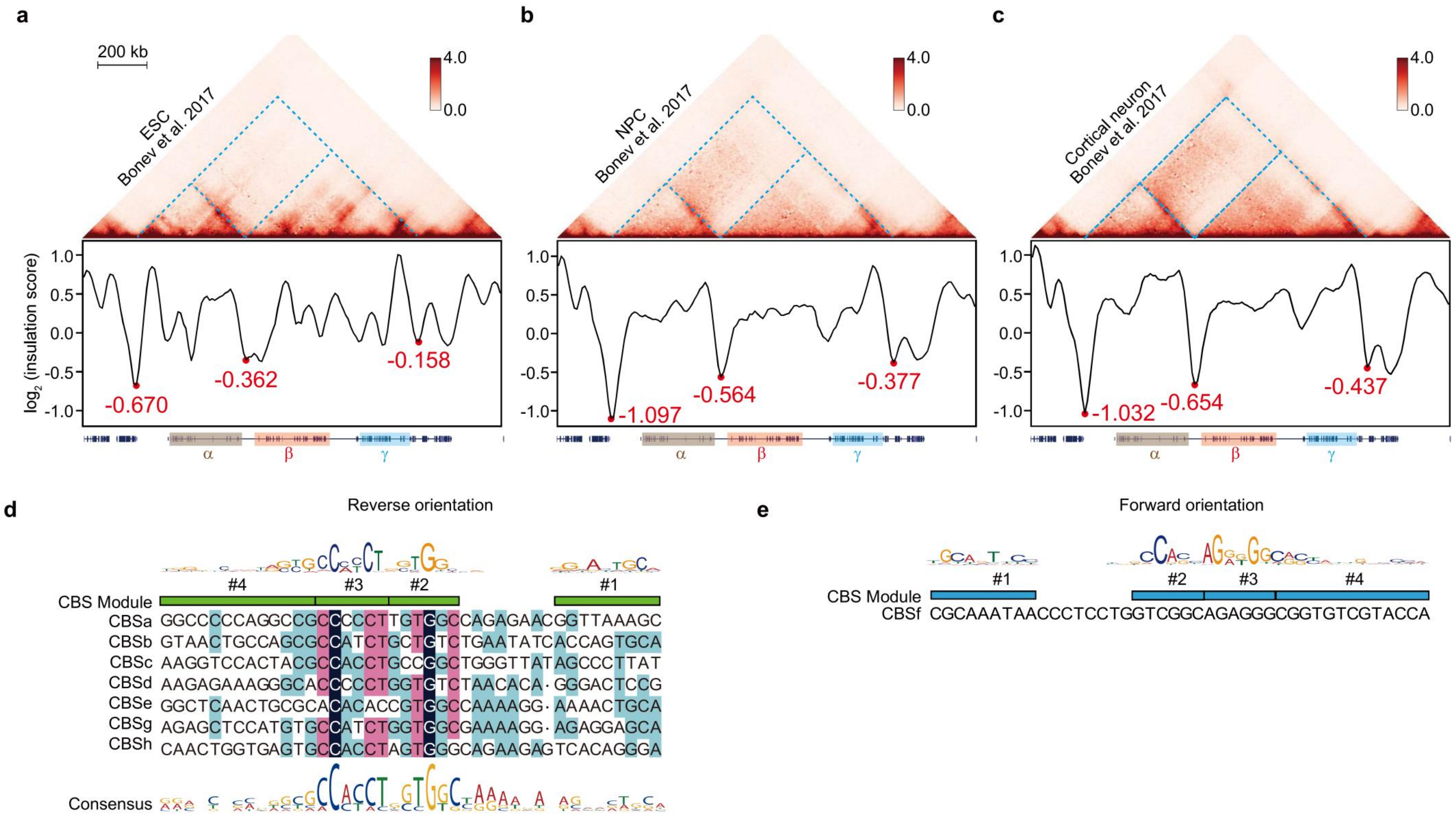

Supplementary Fig. 1. Organization of the three *Pcdh* gene clusters into a large megabase-sized superTAD during mouse neocortical development. a-**c** Hi-C maps of the three *Pcdh* clusters and its flanking regions in mouse ESCs (**a**), NPCs (**b**), and cortical neurons (**c**) obtained during *in vitro* differentiation of ES cells[1], showing that the *Pcdh* superTAD comprised of *Pcdh*α and *Pcdh*$\beta\gamma$ TADs is already established in the mouse ES cells and reinforced during neural development. Insulation score values were shown under the HiC maps. Red dots represent the local minima of the insulation score at the *Pcdh*α and *Pcdh*$\beta\gamma$ TAD boundaries. **d** Alignment of seven reverse-oriented CBS elements (*CBSa-e, CBSg, CBSh*) of the downstream boundary of *Pcdh*$\beta\gamma$ TAD. **e** Sequence of the single forward-oriented *CBSf* element of the downstream boundary of *Pcdh*$\beta\gamma$ TAD.

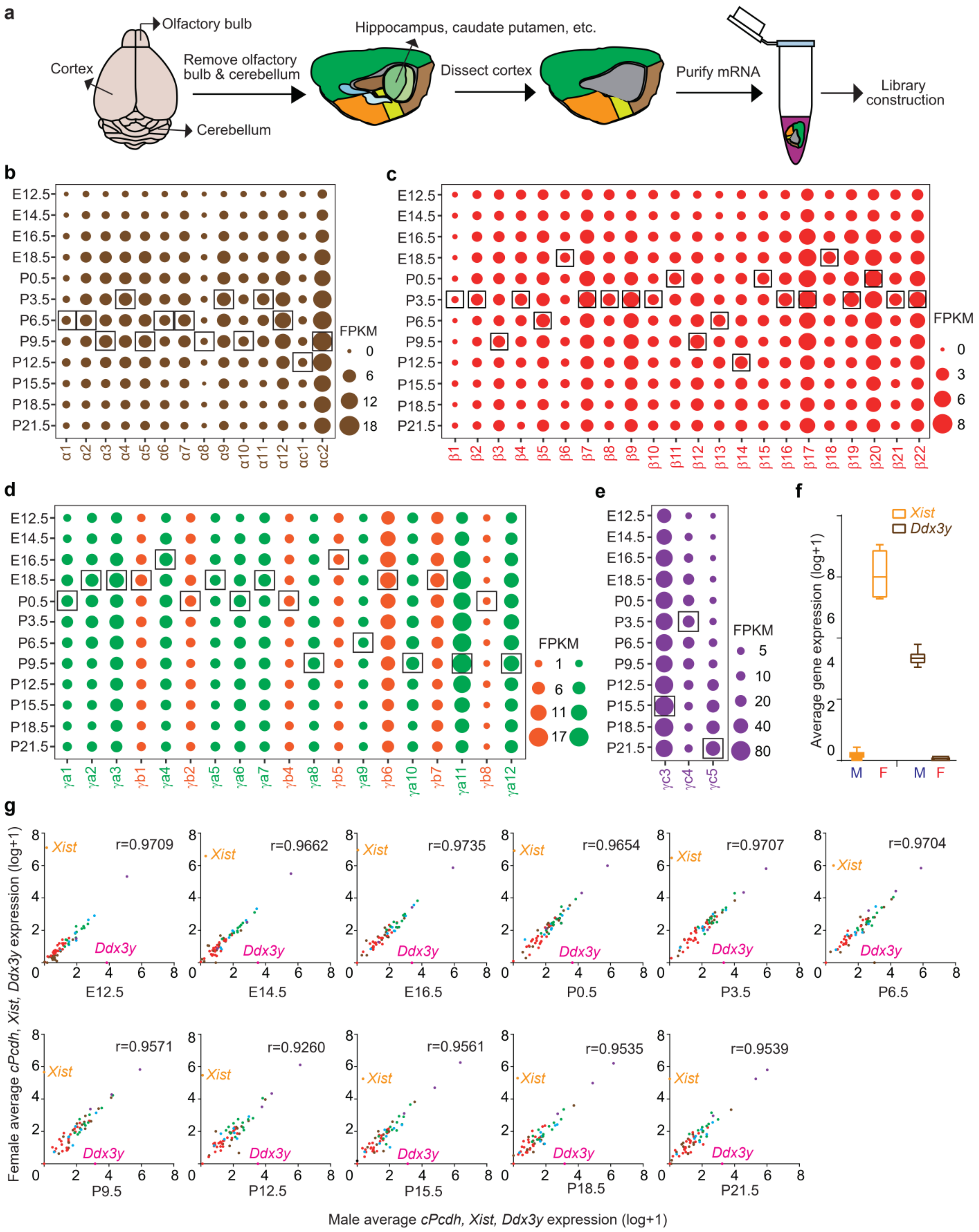

Supplementary Fig. 2. Expression patterns of the *cPcdh* genes during mouse neocortical development. a Schematics of the procedure for generating RNA-seq libraries of the mouse neocortex. Neocortex was microdissected from the whole brain by removing olfactory bulb and other tissues and lysed in Trizol for mRNA purification and library construction. **b**-**e** Expression patterns of members of the *Pcdh*α (**b**), *Pcdh*β (**c**), and *Pcdh*γ (**d**,**e**) clusters during mouse neocortical development. Dot size indicates the expression levels. The outlined dots indicate the peak expression level of each *cPcdh* member at a specific developmental stage. Expression levels were measured by RNA-seq. **f** Normalized expression levels of gender markers of *Ddx3y* and *Xist* between male and female neocortices at P0.5. **g** No gender bias in expression patterns of the *cPcdh* genes. Correlation between male and female *cPcdh* expression levels at all analyzed developmental stages in the mouse neocortex. Pearson correlation coefficients are indicated on the right corner. The Y-chromosome gene *Ddx3y* and X-chromosome gene *Xist* are shown to indicate male and female, respectively. Source data are provided as a Source Data file.

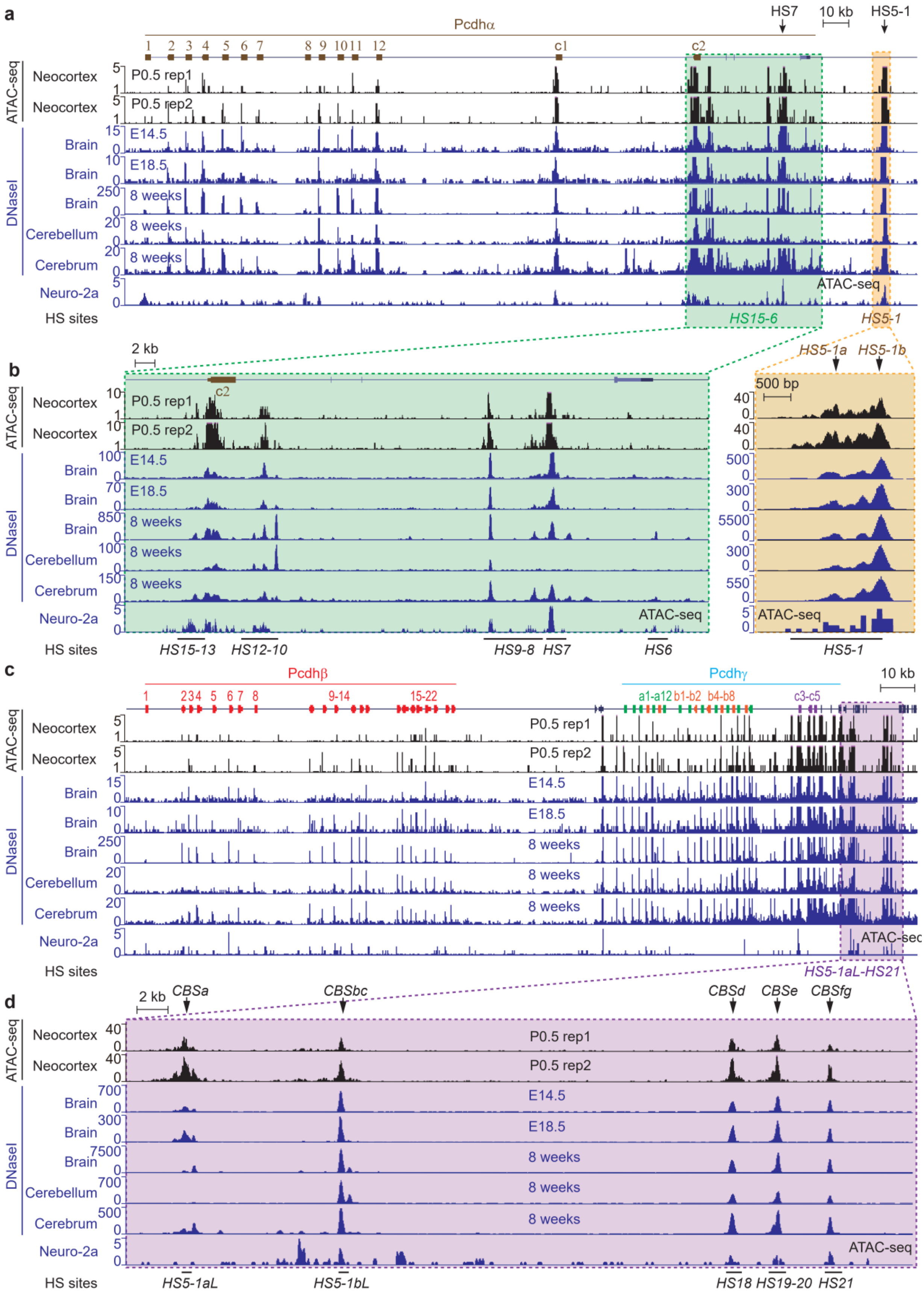

Supplementary Fig. 3. ATAC-seq profiles of the three *Pcdh* gene clusters in mouse newborn neocortex compared to DNaseI-seq profiles in mouse neural tissues. a ATAC-seq profiles of the *Pcdhα* gene cluster in the P0.5 mouse neocortex compared to publicly available DNaseI-seq profiles in the whole brains of E14.5 (GSM1014197), E18.5 (GSM1014184), and 8-week-old (GSM1003823) mice, the cerebellar (GSM1014164) and cerebral (GSM1014168) tissues of 8-week-old mice, and ATAC-seq profiles in the Neuro-2a cell line (GSM2859282). **b** Close-up of ATAC-seq and DNaseI-seq profiles at *HS1-15* sites highlighted in **a**. **c** ATAC-seq profiles of the *Pcdhβγ* clusters in the P0.5 mouse neocortex compared to DNaseI-seq profiles in the whole brains of E14.5, E18.5, and 8-week-old mice, the cerebellar and cerebral tissues of 8-week-old mice, and ATAC-seq profiles in the Neuro-2a cell line. **d** Close-up of ATAC-seq and DNaseI-seq profiles at *HS5-1aL*, *HS5-1bL,* and *HS18-21* highlighted in **c**. CBS elements were indicated above.

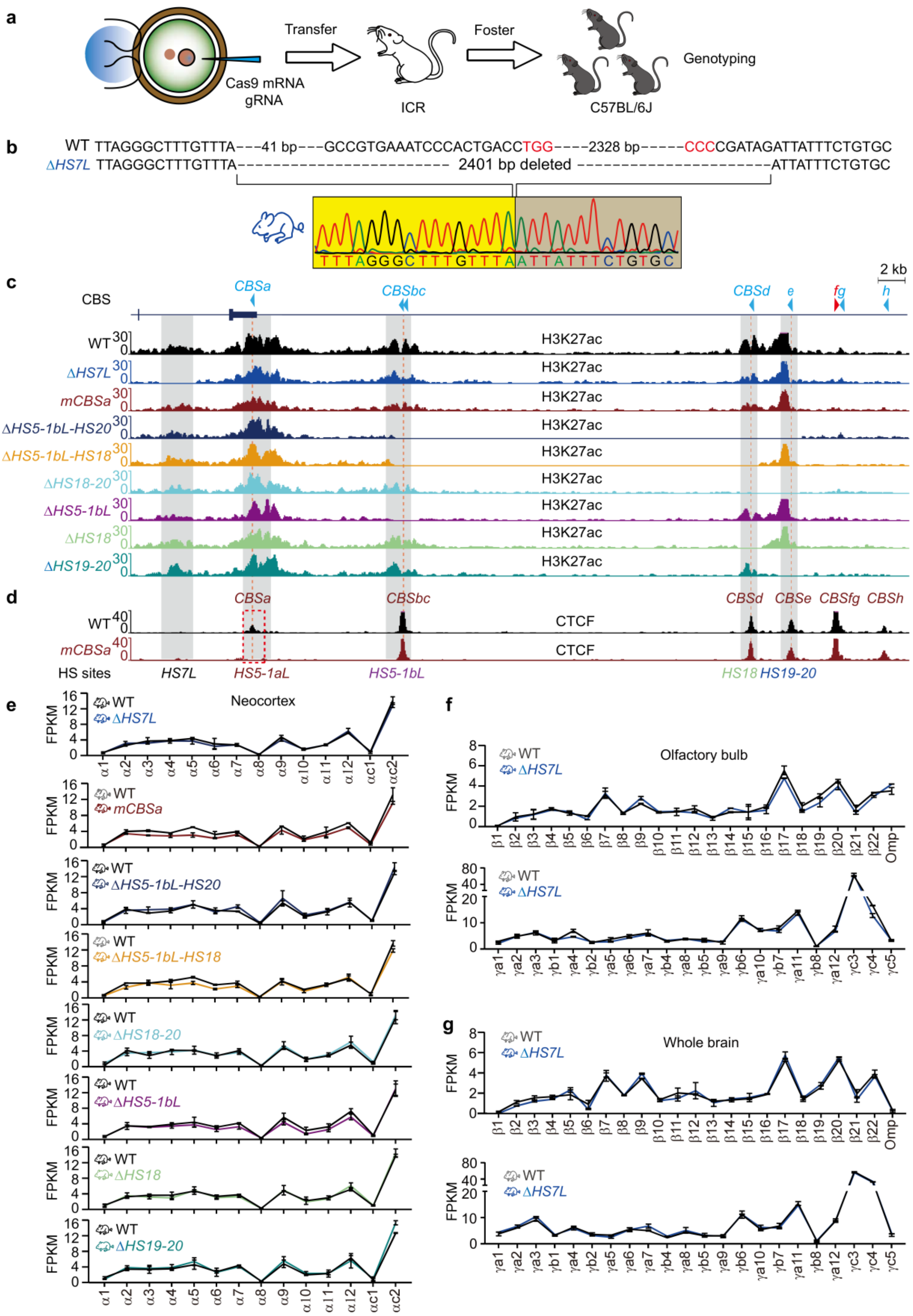

Supplementary Fig. 4. Mouse genetics of a repertoire of *cPcdh cis*-regulatory elements. a Schematics of the procedure for generating CRISPR/Cas9-based DNA fragment targeting mice. Zygotes from E0.5 C57BL/6J mice were microinjected with a solution containing Cas9 mRNA and dual sgRNAs targeting each fragment and transplanted into the oviducts of the pseudo-pregnant ICR female mice. The produced chimeric F0 mice were screened for targeted deletions and crossed with wildtype C57BL/6J mice to generate heterozygous F1 mice. F1 mice were genotyped and crossed to generate F2 homozygous mice. **b** Genotyping of *HS7L*-deleted (Δ*HS7L*) homozygous mice by Sanger sequencing. **c** H3K27ac ChIP-seq profiles of the clustered CTCF TAD boundary in each mutant mouse line. **d** ChIP-seq profiles showing the loss of CTCF enrichments in *CBSa*-mutant (*mCBSa*) mice. **e** RNA-seq showing no significant alteration of *Pcdh*α expression levels in all of the targeted mice. **f**-**g** RNA-seq showing no alteration of *Pcdh* β and γ expression levels in the olfactory bulb (**f**) and whole brain (**g**) tissues of the Δ*HS7L* mice. Omp, olfactory marker protein. Data as mean ± SD; two-tailed Student's *t* test. For each mouse line, n = 4 (**e**) or 2 (**f**,**g**) biologically independent samples; for their WT littermate controls, n = 2. Source data are provided as a Source Data file.

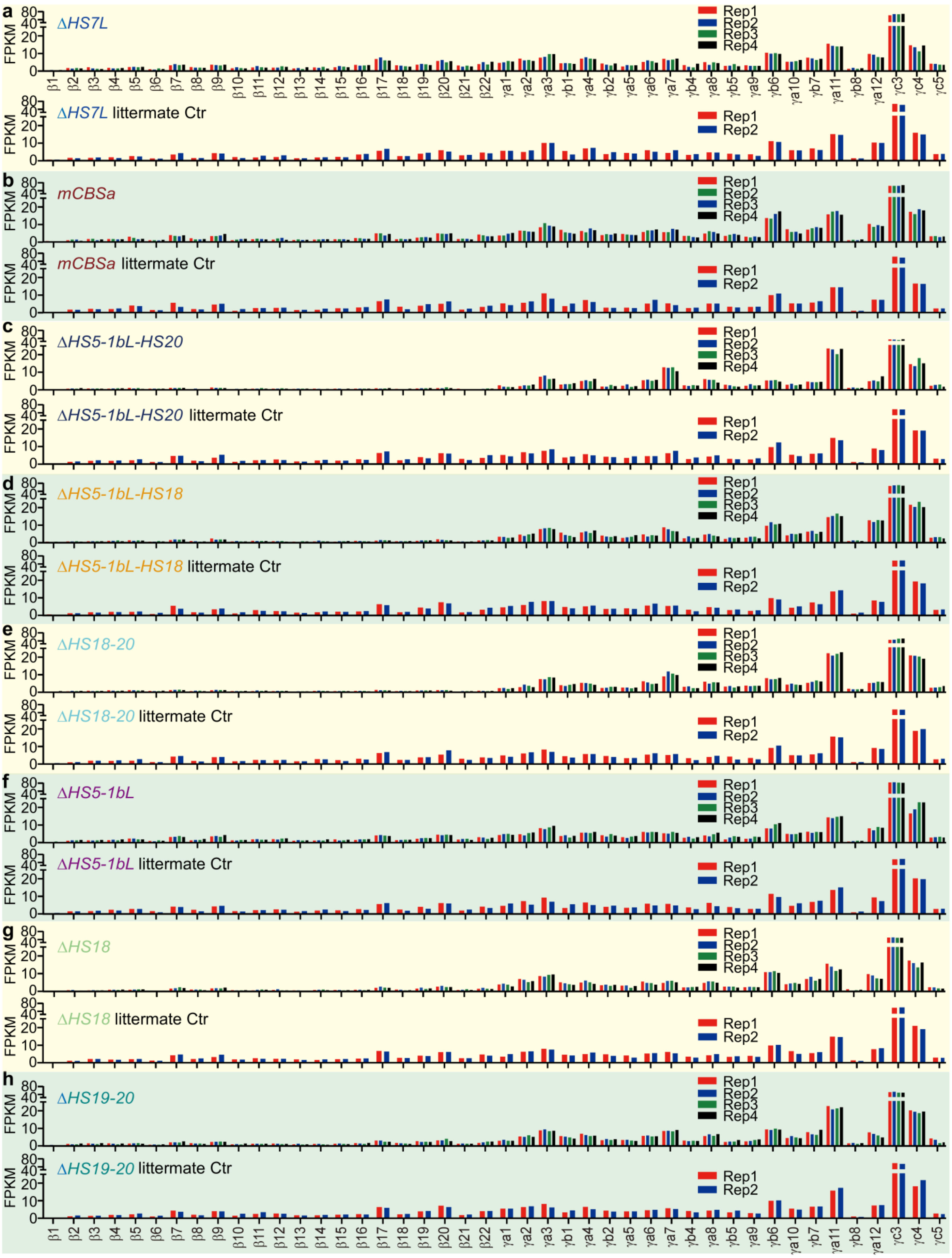

Supplementary Fig. 5. Replicates of RNA-seq levels of the *Pcdhβγ* genes. a-h Expression levels of replicates of RNA-seq experiments for Δ*HS7L* (**a**), m*CBSa* (**b**), Δ*HS5-1bL-HS20* (**c**), Δ*HS5-1bL-HS18* (**d**), Δ*HS18-20* (**e**), Δ*HS5-1bL* (**f**), Δ*HS18* (**g**), or Δ*HS19-20* (**h**) mice compared to their wild-type littermates. Expression levels were based on the FPKM values. Data as mean ± SD. * P < 0.05, ** P < 0.01, *** P < 0.001. For each mouse line, two wild-type replicates and four mutant replicates were performed.

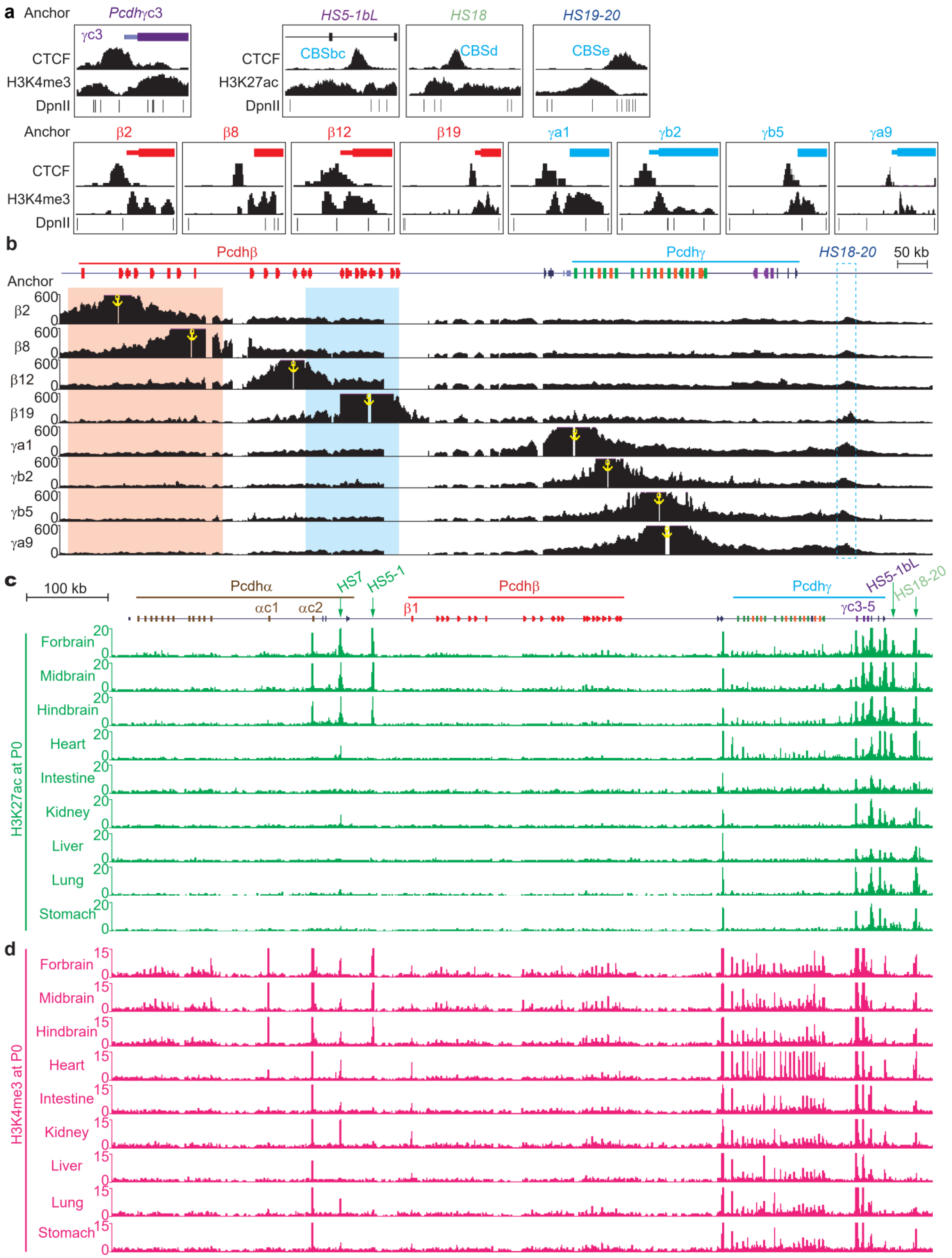

Supplementary Fig. 6. Chromosome conformation and tissue specificity of the *Pcdh* enhancers. a Schematic diagrams showing the locations of each 4C anchor. **b** 4C profiles using a repertoire of *Pcdhβγ* promoters as anchors showing their close contacts with *HS18-20*. **c**,**d** H3K27ac (**c**) and H3K4me3 (**d**) ChIP-seq profiles of histone marks in neural and non-neural tissues in P0 mice[2].

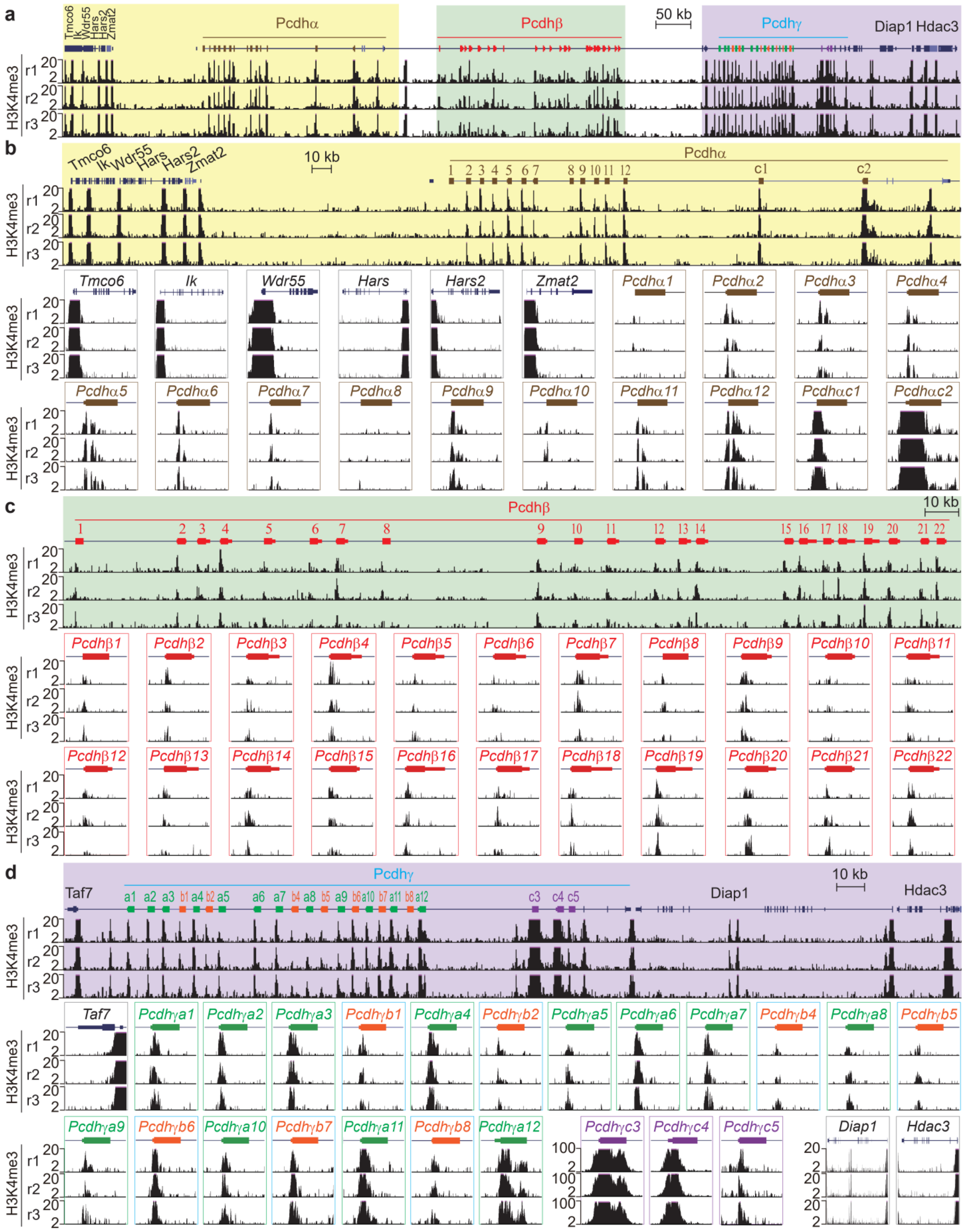

Supplementary Fig. 7. Enrichments of the active mark of H3K4me3 at the promoter region of each member of the three *Pcdh* gene clusters in the mouse brain. a H3K4me3 ChIP-seq profiles at the *cPcdh* locus and its flanking regions with three replicates. **b**-**d** Close-up of H3K4me3 profiles of the *Pcdhα* (**b**), *Pcdhβ* (**c**), or *Pcdhγ* (**d**) gene cluster.

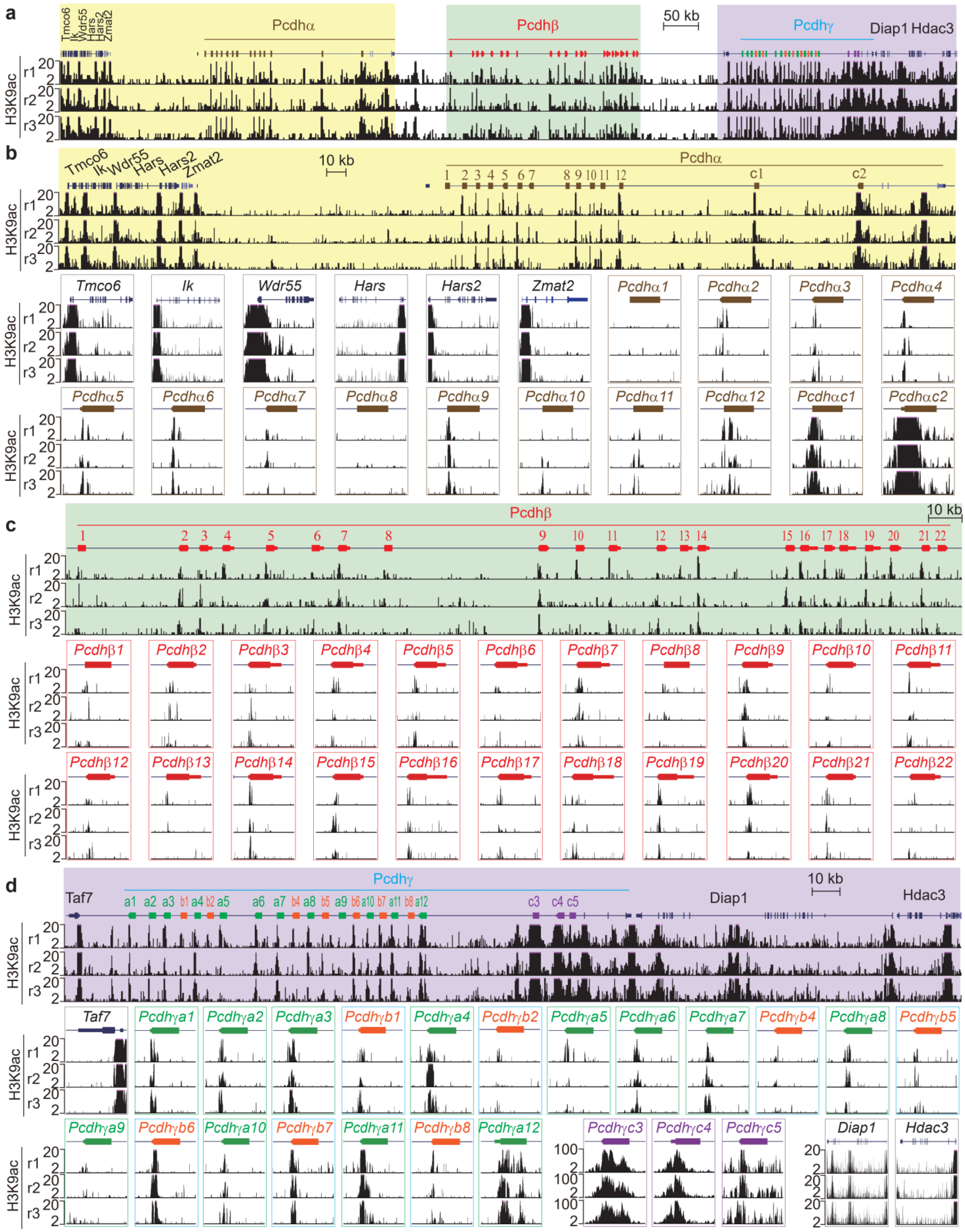

Supplementary Fig. 8. Enrichments of the active mark of H3K9ac at the *cPcdh* promoters and enhancers. a H3K9ac ChIP-seq profiles at the three *Pcdh* gene clusters and their flanking regions with three replicates. **b-d** Close-up of H3K9ac profiles of the *Pcdhα* (**b**), *Pcdhβ* (**c**), or *Pcdhγ* (**d**) gene cluster.

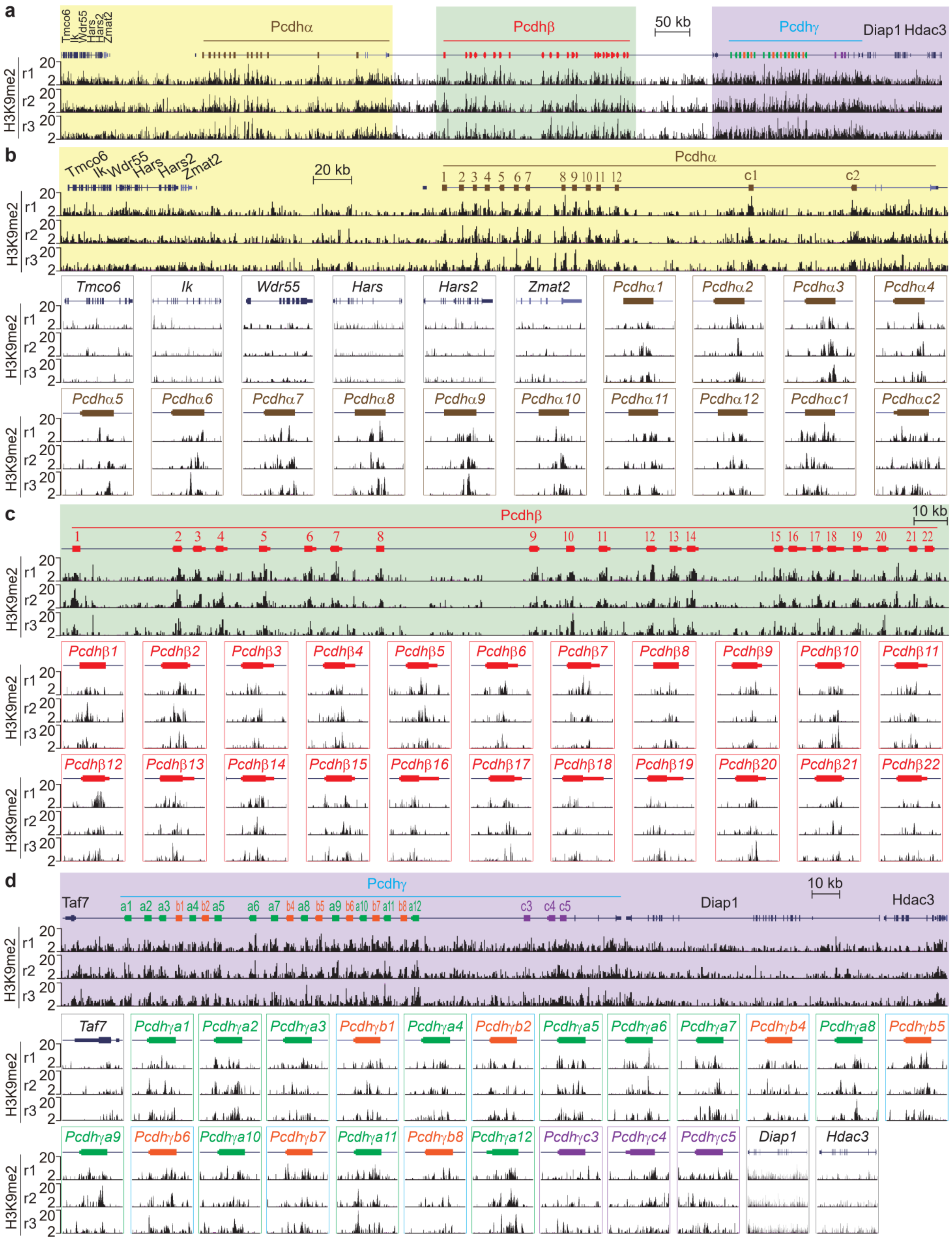

Supplementary Fig. 9. Enrichments of the heterochromatin mark of H3K9me2 at each member of the three *Pcdh* gene clusters in the brain. a H3K9me2 ChIP-seq profiles at the *cPcdh* locus and its flanking regions with three replicates. **b-d** Close-up of H3K9me2 profiles of the *Pcdhα* (**b**), *Pcdhβ* (**c**), or *Pcdhγ* (**d**) gene cluster, showing the correlation of each c*Pcdh* variable exon with H3K9me2 compared to the flanking genes.

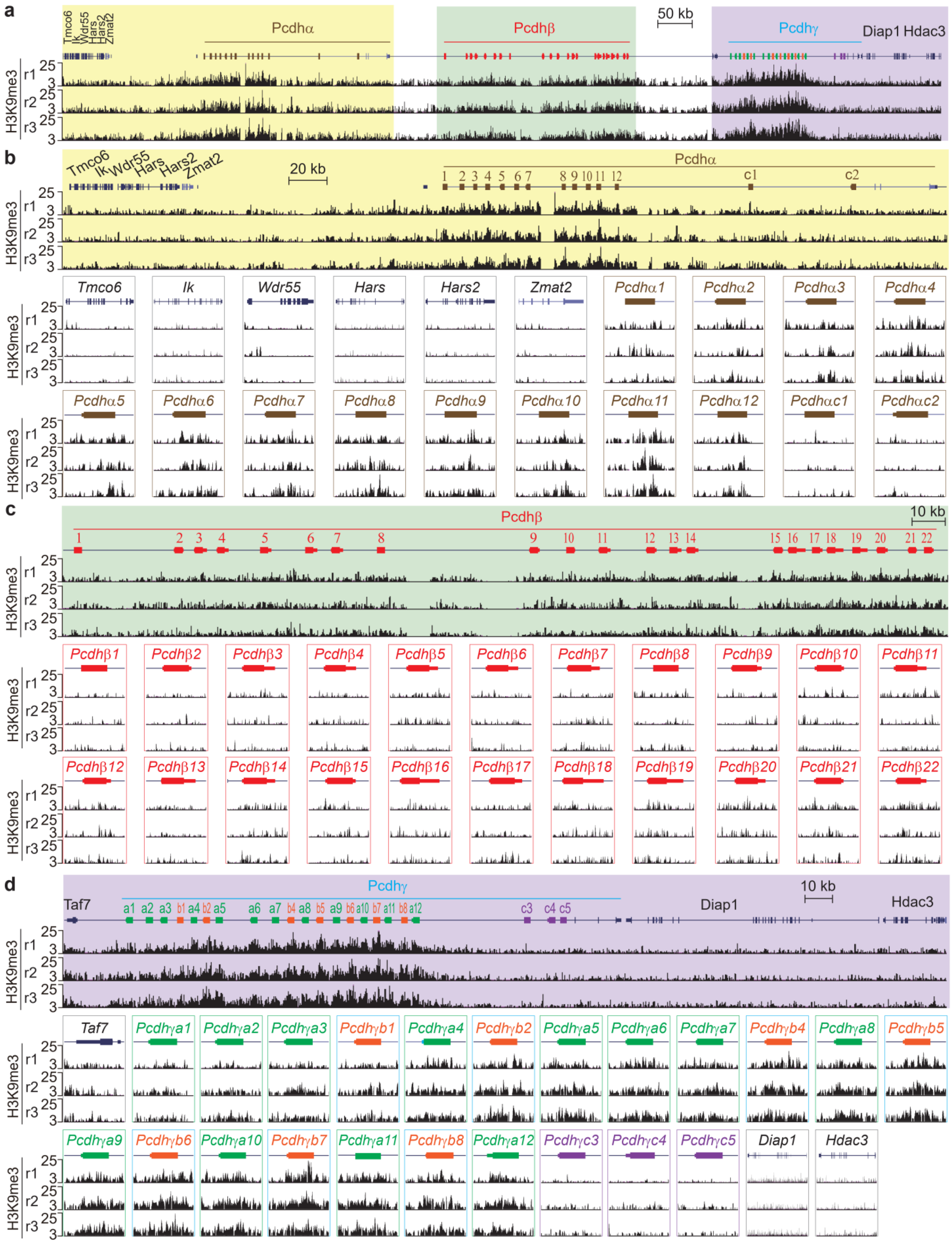

Supplementary Fig. 10. Enrichments of the heterochromatin mark of H3K9me3 at the monoallelic, but not biallelic, *cPcdh* genes in the brain. a H3K9me3 ChIP-seq profiles at the *Pcdh* locus and the flanking regions with three replicates. **b**-**d** Close-up of H3K9me3 profiles of the *Pcdhα* (**b**), *Pcdhβ* (**c**), or *Pcdhγ* (**d**) gene cluster, showing correlation of each monoallelic *Pcdh* variable exon with H3K9me3. Note the absence of H3K9me3 mark in the five biallelic C-type *Pcdh* variable exons.

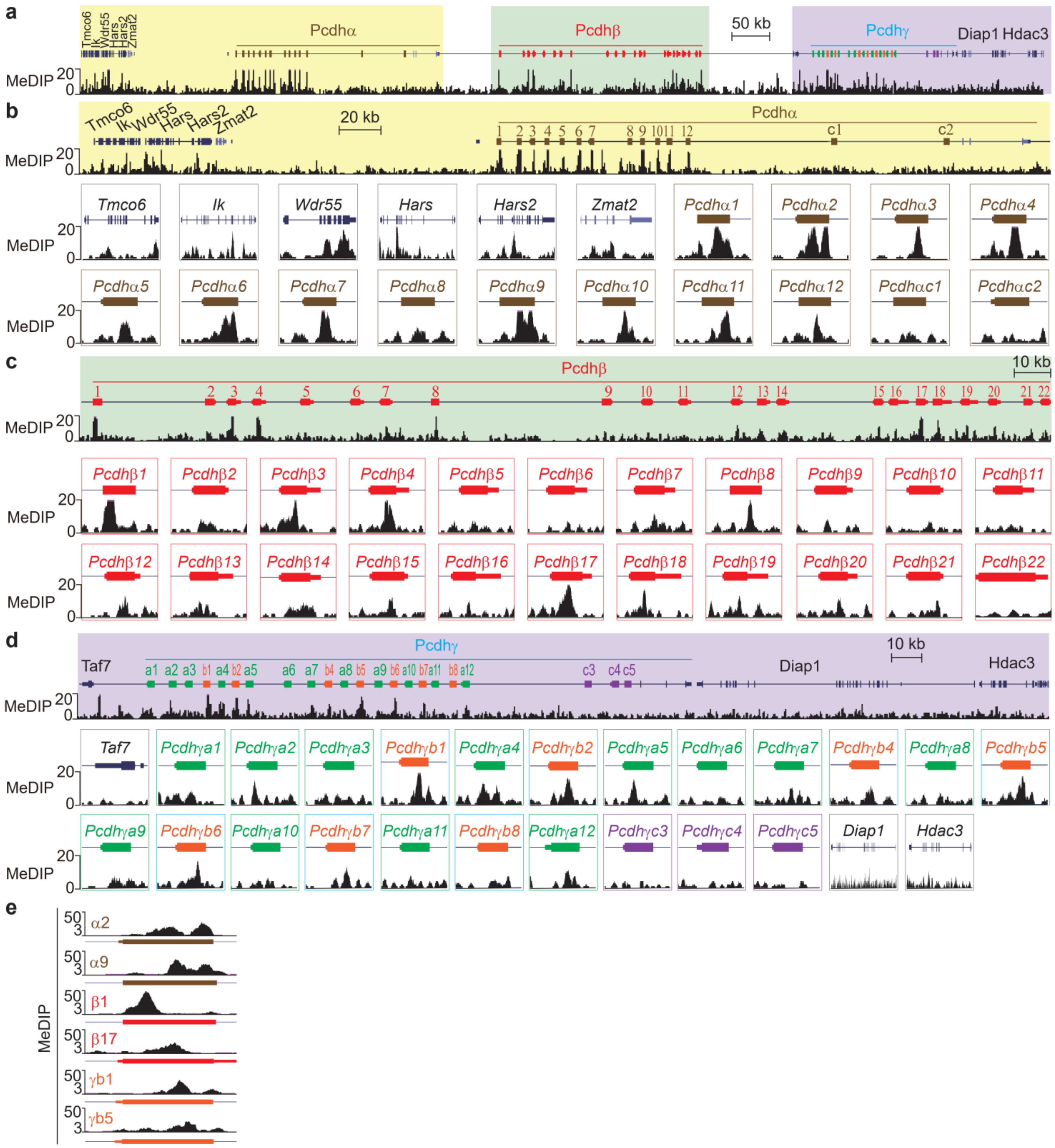

Supplementary Fig. 11. Enrichments of DNA methylation at the *cPcdh* genes in the brain. a MeDIP-seq profile at the three *Pcdh* clusters and their flanking regions, analyzed from our previous data[3]. **b-d** Close-up of MeDIP-seq profiles of the *Pcdhα* (**b**), *Pcdhβ* (**c**), or *Pcdhγ* (**d**) gene cluster, showing the strong DNA methylation at the 3' end of monoallelic *Pcdhα* variable exons and moderate DNA methylation at most *Pcdhβγ* variable exons. **e** Note the methylation at the 5' end of *Pcdhβ1* compared to methylation at the 3' end of other *cPcdh* genes.

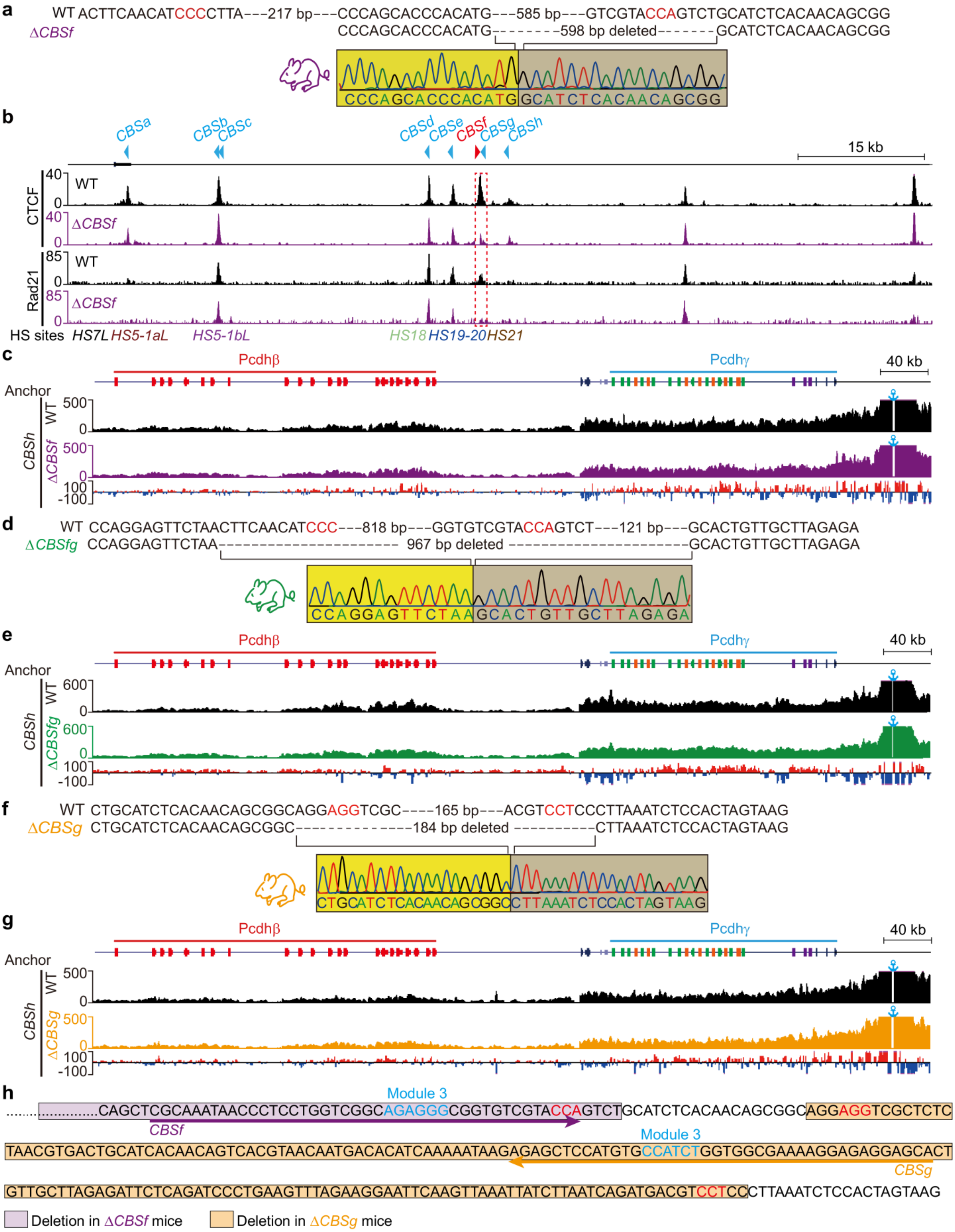

Supplementary Fig. 12. Increased chromatin interactions between *Pcdhβγ* and the downstream *CBSh* region upon *CBSf* or *CBSfg* deletion. **a**,**d**,**f** Genotyping of Δ*CBSf* (**a**), Δ*CBSfg* (**d**), or Δ*CBSg* (**f**) homozygous mice by Sanger sequencing. **b** CTCF and Rad21 ChIP-seq profiles centered on *CBSf* in the Δ*CBSf* mice compared to their WT littermates. **c**,**e**,**g** 4C profiles of the Δ*CBSf* (**c**), Δ*CBSfg* (**e**), or Δ*CBSg* (**g**) mouse neocortex using *CBSh* as an anchor. **h** Targeting sites for CRIPSR deletion of *CBSf* or *CBSg*.

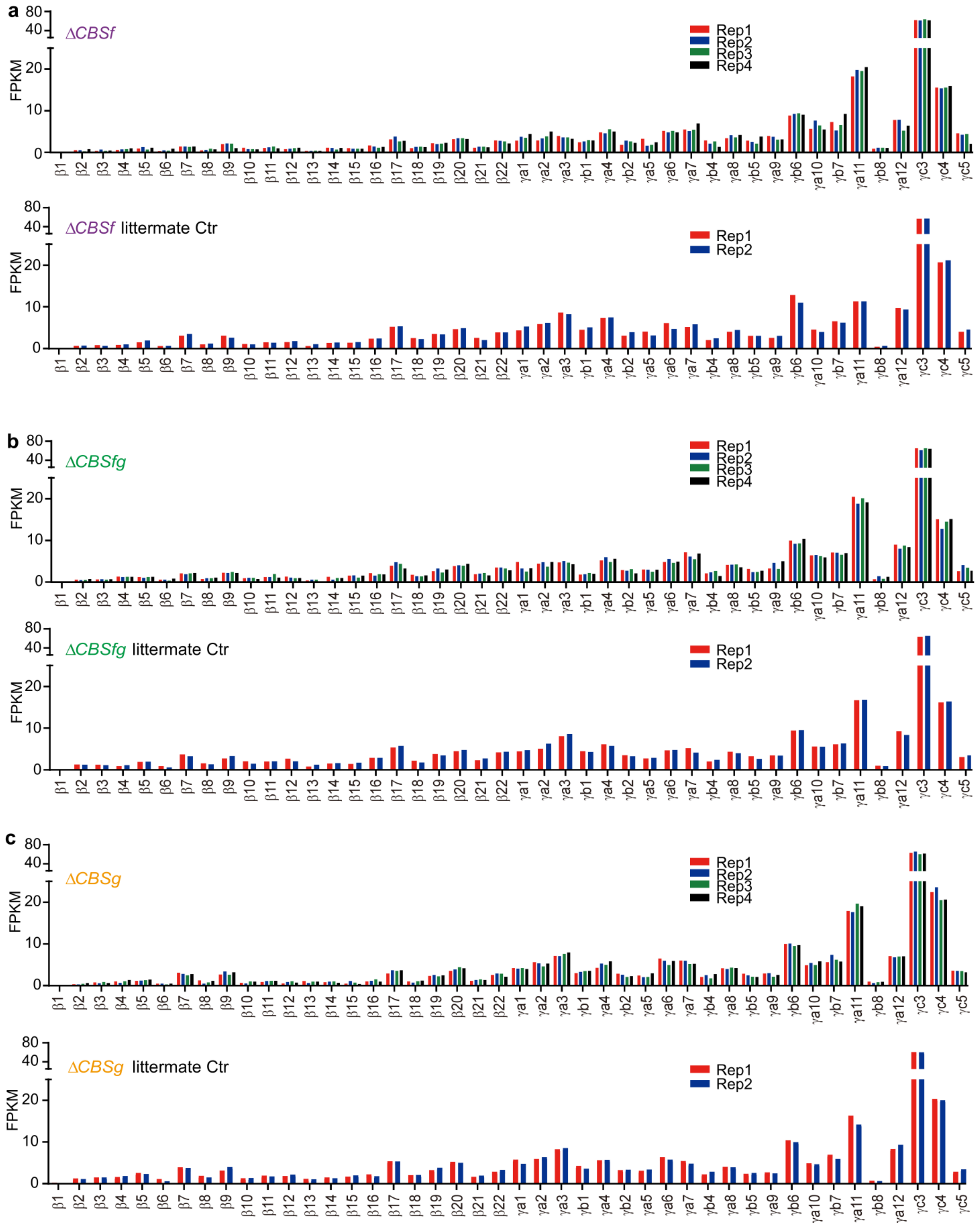

Supplementary Fig. 13. Replicates of RNA-seq experiments of the forward-oriented CBS-deletion mice. a-**c** Expression levels of RNA-seq replicates of microdissected neocortical tissues from the Δ*CBSf* (**a**), Δ*CBSfg* (**b**) or Δ*CBSg* (**c**) homozygous mice compared to their wild-type littermates. Expression levels were based on the FPKM values. Data as mean ± SD. * $P < 0.05$, ** $P < 0.01$, *** $P < 0.001$. For each mutant mouse line, two wild-type replicates and four deletion replicates were performed.

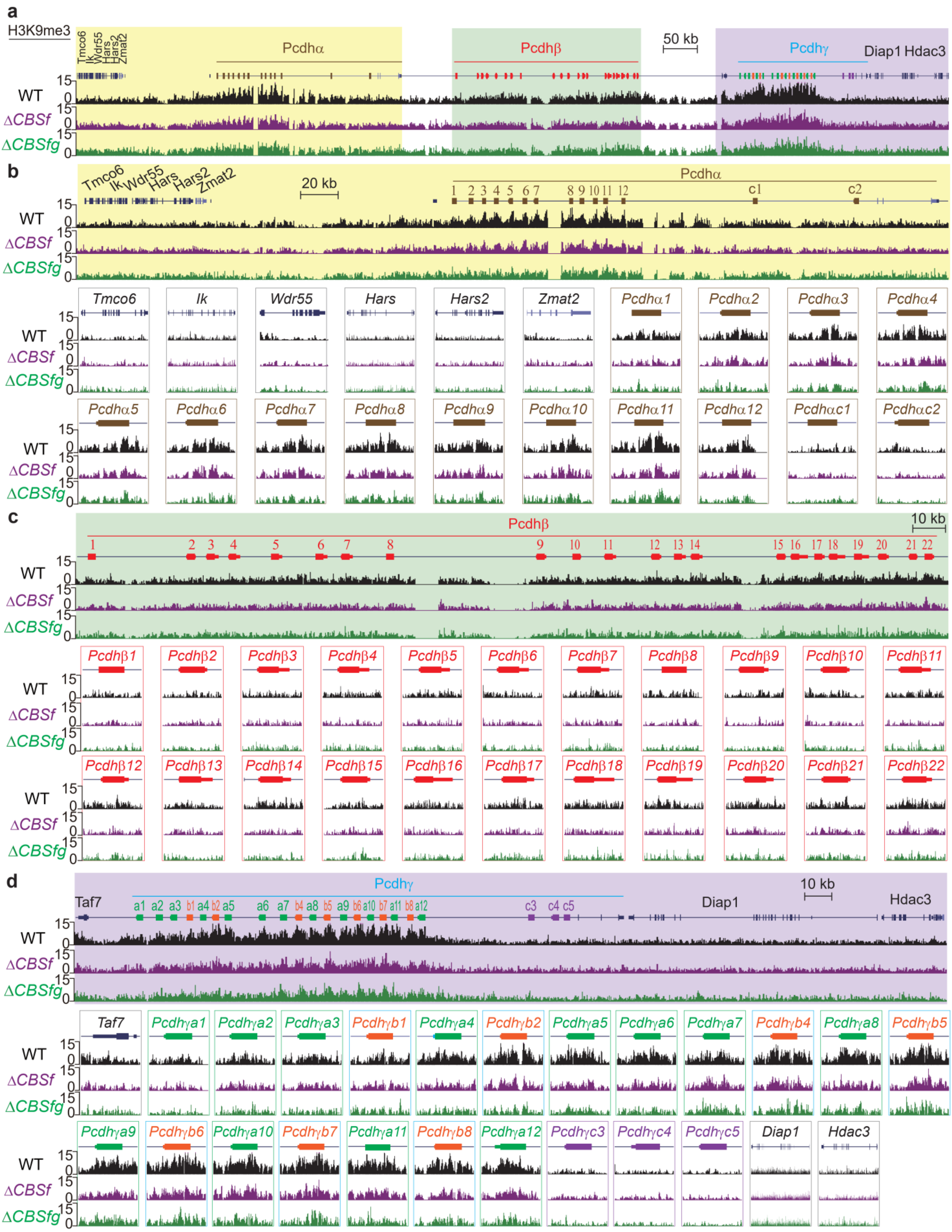

Supplementary Fig. 14. Decreased enrichments of the heterochromatin mark of H3K9me3 at the monoallelic *cPcdh* genes in the brain upon *CBSf* or *CBSfg* knockout. a H3K9me3 ChIP-seq profiles at the *Pcdh* locus and the flanking regions in Δ*CBSf* or Δ*CBSfg* homozygous mice compared to WT mice. **b**-**d** Close-up of H3K9me3 profiles at the *Pcdhα* (**b**), *Pcdhβ* (**c**), or *Pcdhγ* (**d**) gene cluster, showing decreased enrichments of H3K9me3 at each monoallelic *Pcdh* variable exon.

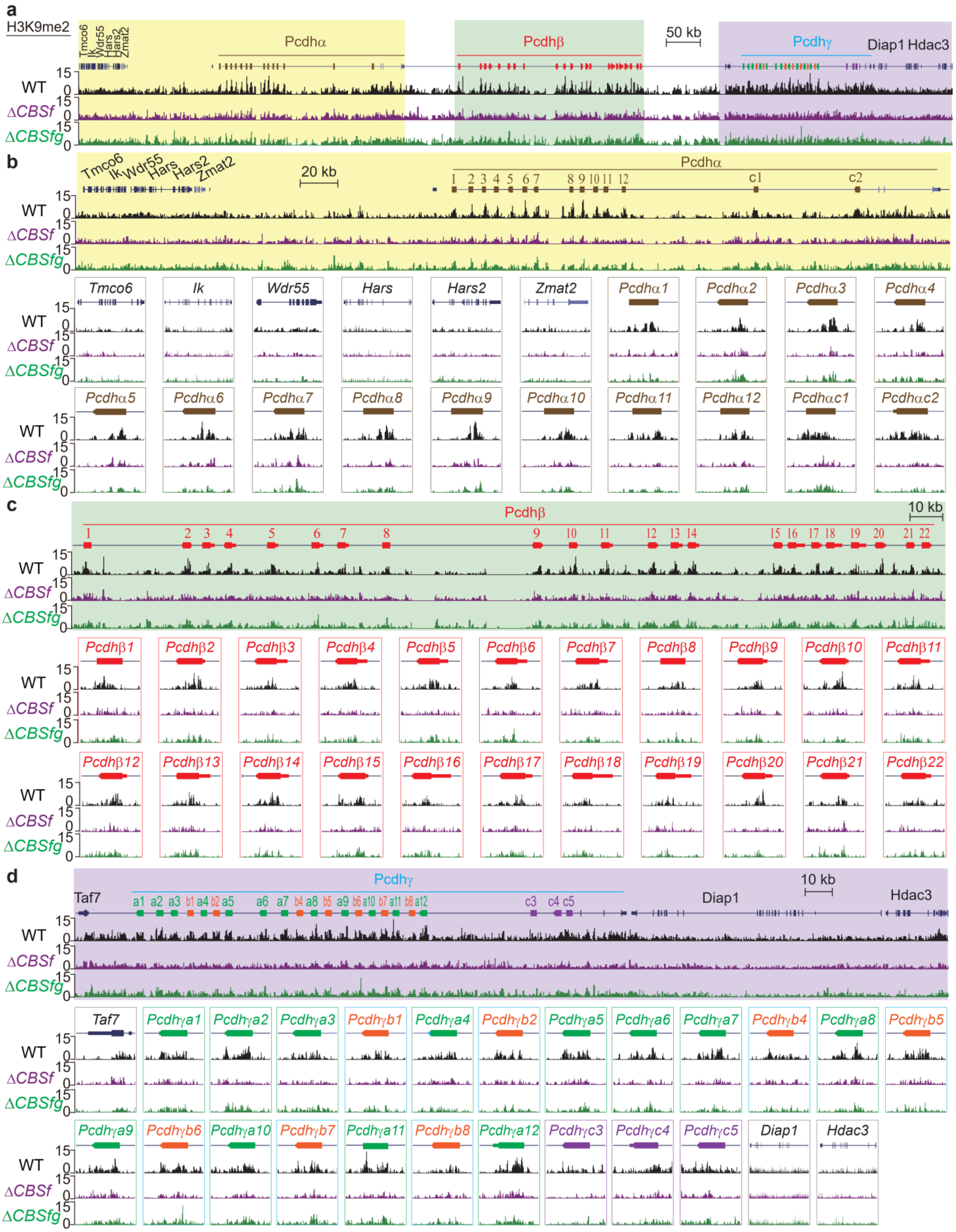

Supplementary Fig. 15. Decreased enrichments of the heterochromatin mark of H3K9me2 at the *cPcdh* genes in the brain upon *CBSf* or *CBSfg* knockout. a H3K9me2 ChIP-seq profiles at the *cPcdh* locus and its flanking regions in Δ*CBSf* or Δ*CBSfg* homozygous mice compared to WT mice. **b**-**d** Close-up of H3K9me2 profiles at the *Pcdhα* (**b**), *Pcdhβ* (**c**), or *Pcdhγ* (**d**) gene cluster, showing decreased enrichments of H3K9me2 at each c*Pcdh* variable exon.

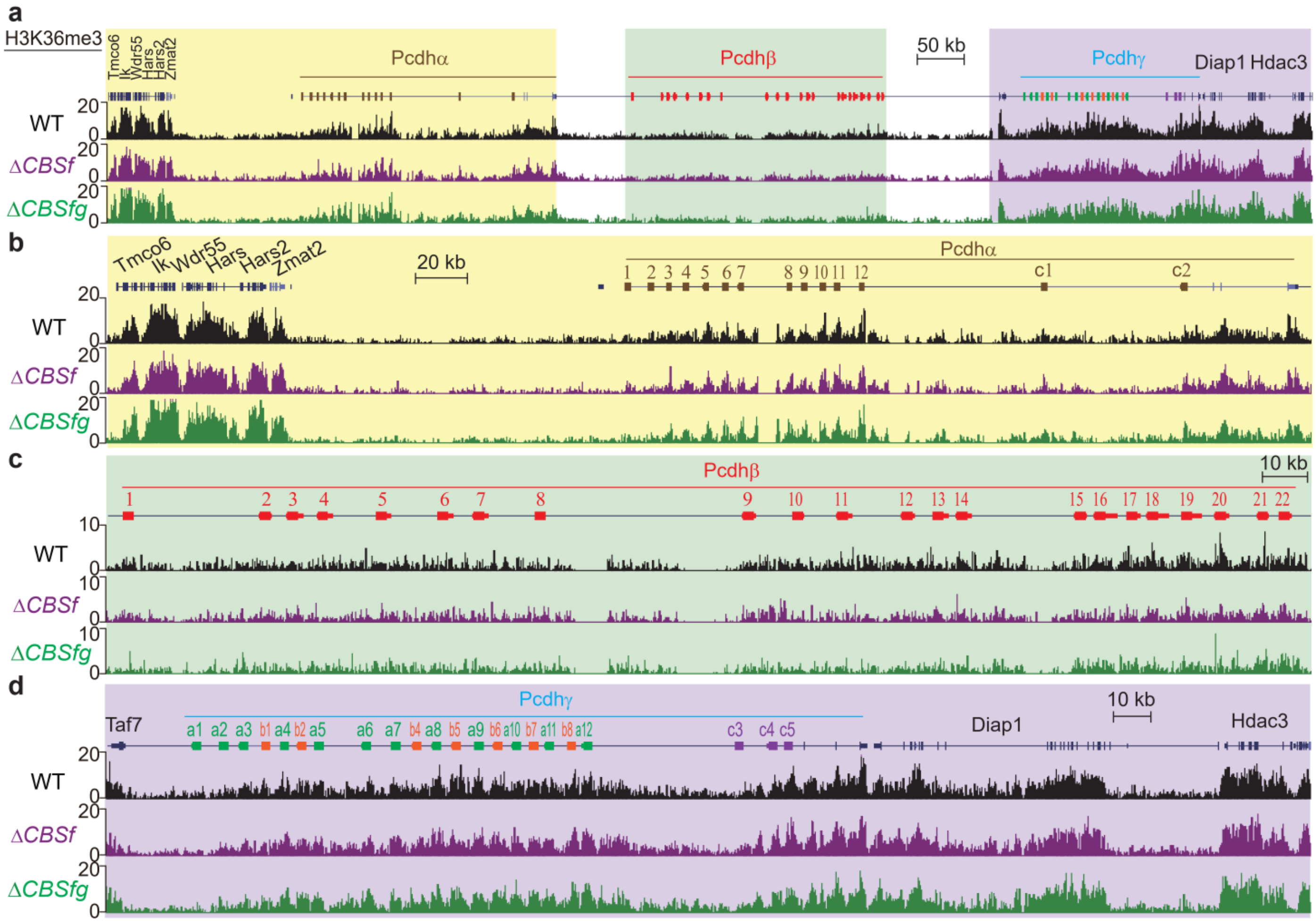

Supplementary Fig. 16. Enrichments of the active mark of H3K36me3 at the *cPcdh* genes in the brain of *CBSf* or *CBSfg* knockout mice. a H3K36me3 ChIP-seq profiles at the *cPcdh* locus and its flanking regions. **b**-**d** Close-up of the H3K36me3 profiles at the *Pcdhα* (**b**), *Pcdhβ* (**c**), or *Pcdhγ* (**d**) gene cluster.

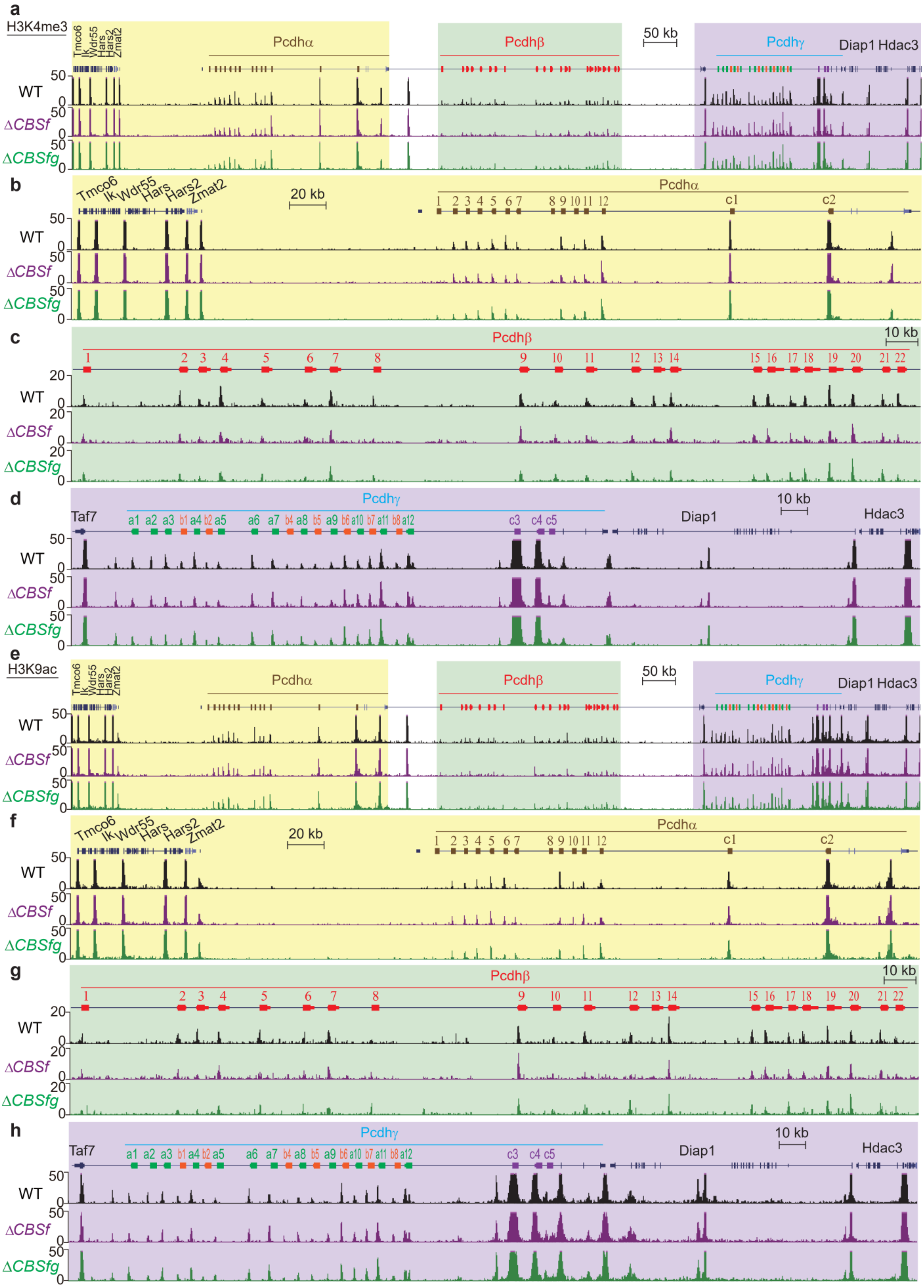

Supplementary Fig. 17. Enrichments of the active mark of H3K4me3 or H3K9ac at the *cPcdh* genes in the brain of *CBSf* or *CBSfg* knockout mice. a H3K4me3 ChIP-seq profiles at the *cPcdh* locus and its flanking regions. **b**-**d** Close-up of the H3K4me3 profiles at the *Pcdhα* (**b**), *Pcdhβ* (**c**), or *Pcdhγ* (**d**) gene cluster. **e** H3K9ac ChIP-seq profiles at the *cPcdh* locus and its flanking regions. **f**-**h** Close-up of the H3K9ac profiles at the *Pcdhα* (**f**), *Pcdhβ* (**g**), or *Pcdhγ* (**h**) gene cluster.

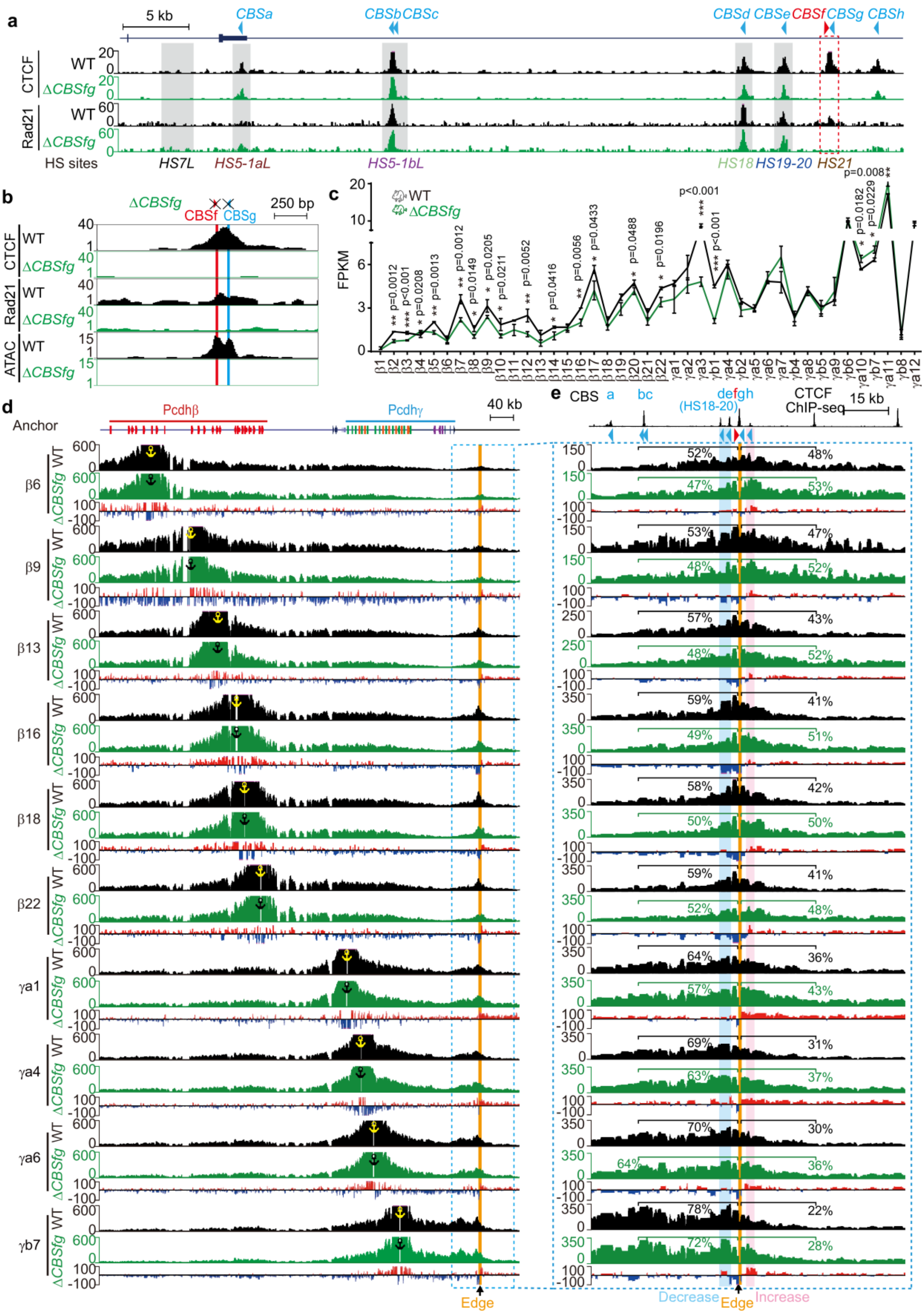

Supplementary Fig. 18. Combined deletion of both *CBSf* and *CBSg* elements results in a phenotype similar to that of the *CBSf* deletion. a CTCF and Rad21 ChIP-seq profiles of the downstream boundary of the *Pcdhβγ* TAD in Δ*CBSfg* mice compared to their wild-type (WT) littermates showing the abolishment of CTCF and Rad21 enrichments upon *CBSfg* knockout. **b** Close-up of the CTCF and Rad21 ChIP-seq as well as ATAC-seq profiles in Δ*CBSfg* mice compared to their WT littermates. **c** RNA-seq of *Pcdhβγ* in Δ*CBSfg* mice compared to their WT littermates showing a significant decrease of the expression of the *Pcdhβγ* clusters. Data as mean ± SD, *$p < 0.05$, **$p < 0.01$, ***$p < 0.001$; two-tailed Student's *t* test. For Δ*CBSfg* mouse line, n = 4 biologically independent samples; for their WT littermate controls, n = 2. Source data are provided as a Source Data file. **d**,**e** 4C profiles using a repertoire of the *Pcdhβγ* promoters as anchors showing increased chromatin interactions beyond the location of *CBSfg* (highlighted in pink, **d** and **e**) and decreased chromatin interactions with *HS18-20* enhancers (highlighted in blue, **e**). Interaction differences (Δ*CBSfg* versus WT) are shown under the 4C profiles. Note a sharp transition edge at the location of the *CBSfg* element.

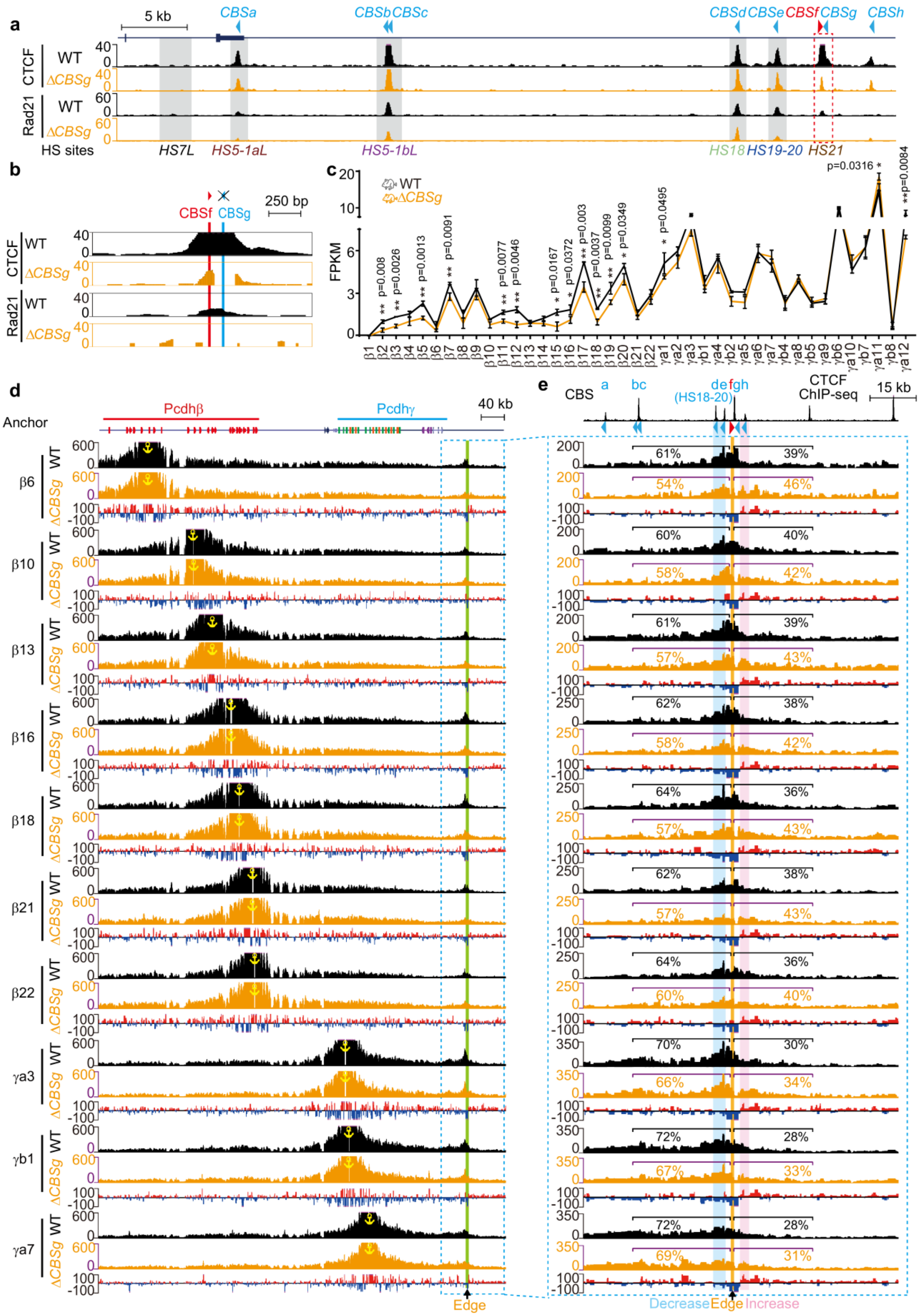

Supplementary Fig. 19. Deletion of *CBSg* results in a slight decrease in *Pcdhβγ* expression. a CTCF and Rad21 ChIP-seq profiles of the *Pcdhβγ* downstream TAD boundary in Δ*CBSg* mice compared to their wild-type (WT) littermates. **b** Close-up of the CTCF and Rad21 ChIP-seq profiles in Δ*CBSg* mice compared to their WT littermates. **c** RNA-seq showing a slight decrease in *Pcdhβγ* expression levels upon *CBSg* deletion. Data as mean ± SD, $*p < 0.05$, $**p < 0.01$, $***p < 0.001$; two-tailed Student's *t* test. For Δ*CBSg* mouse line, n = 4 biologically independent samples; for their WT littermate controls, n = 2. Source data are provided as a Source Data file. **d**,**e** 4C profiles using a repertoire of *Pcdhβγ* promoters as anchors showing decreased chromatin interactions with *HS18-20* enhancers (highlighted in blue, **e**) and increased chromatin interactions beyond *CBSg* (highlighted in pink, **e**). Note a sharp edge for the transition from decrease to increase at the location of *CBSg*. Differences (Δ*CBSg* versus WT) are shown under the 4C profiles.

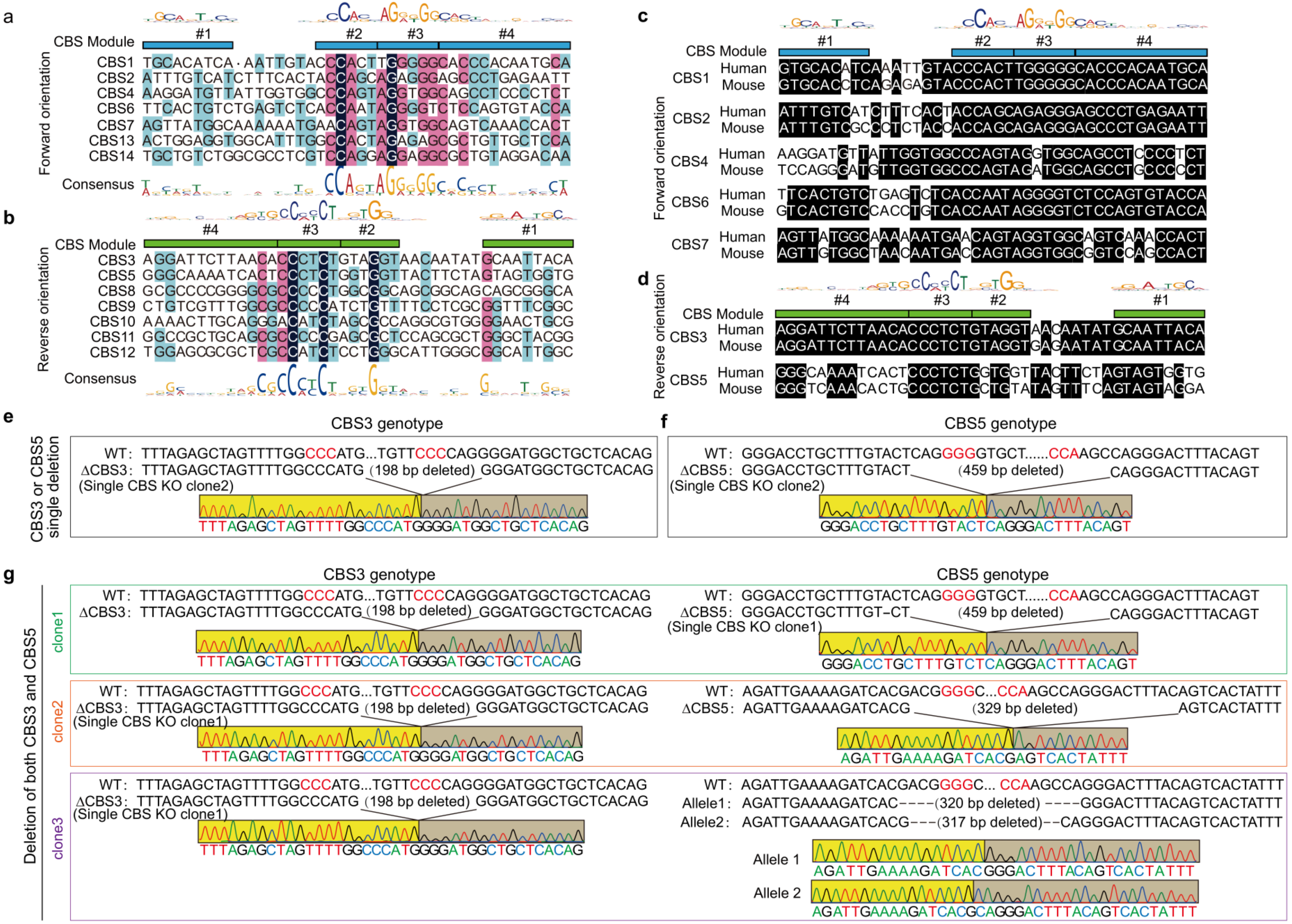

Supplementary Fig. 20. Single or double knockout of outward-oriented CBS elements within the clustered CTCF TAD boundary of the *HOXD* gene cluster. a,**b** Alignments of all the forward (**a**) and reverse (**b**) CBS elements of the *HOXD* C-DOM. **c**,**d** Conservation of the CBS elements of the *HOXD* C-DOM between human and mouse. **e**-**g** Genotyping of Δ*CBS3* (**e**), Δ*CBS5* (**f**) or Δ*CBS3+5* (**g**) single-cell clones by Sanger sequencing.

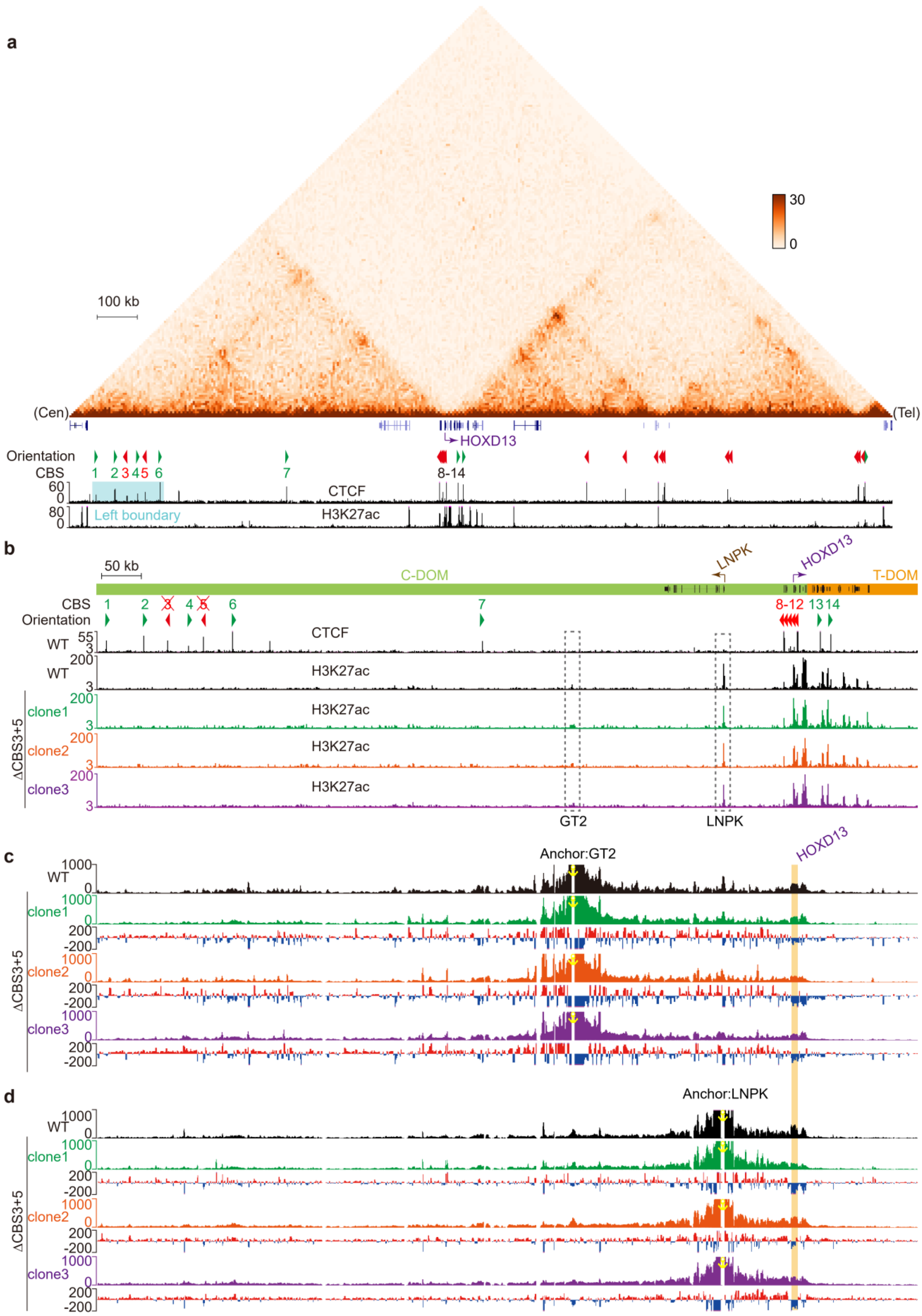

Supplementary Fig. 21. Outward-oriented *CBS* elements of the C-DOM boundary are crucial for *HOXD13* expression. a Hi-C map of the *HOXD* genomic region showing the TAD organization around the *HOXD* locus. The *HOXD* cluster are located between the two regulatory domains: centromeric TAD (C-DOM) and telomeric TAD (T-DOM). Arrowheads indicate CBS elements with orientations. CTCF and H3K27ac ChIP-seq profiles showing that the left boundary (highlighted in blue) of C-DOM is free of H3K27ac marks and comprises six clustered CBS elements (*CBS1-6*), of which *CBS3* and *CBS5* are outward-oriented. **b** H3K27ac ChIP-seq profiles of the C-DOM in the three ΔCBS*3+5* single-cell clones compared to wild-type (WT) clones. **c**,**d** 4C profiles using the H3K27ac-enriched region of *GT2* (**c**) or *NLPK* (**d**) as an anchor, showing decreased interactions with *HOXD13* in three ΔCBS*3+5* single-cell clones compared to WT clones.

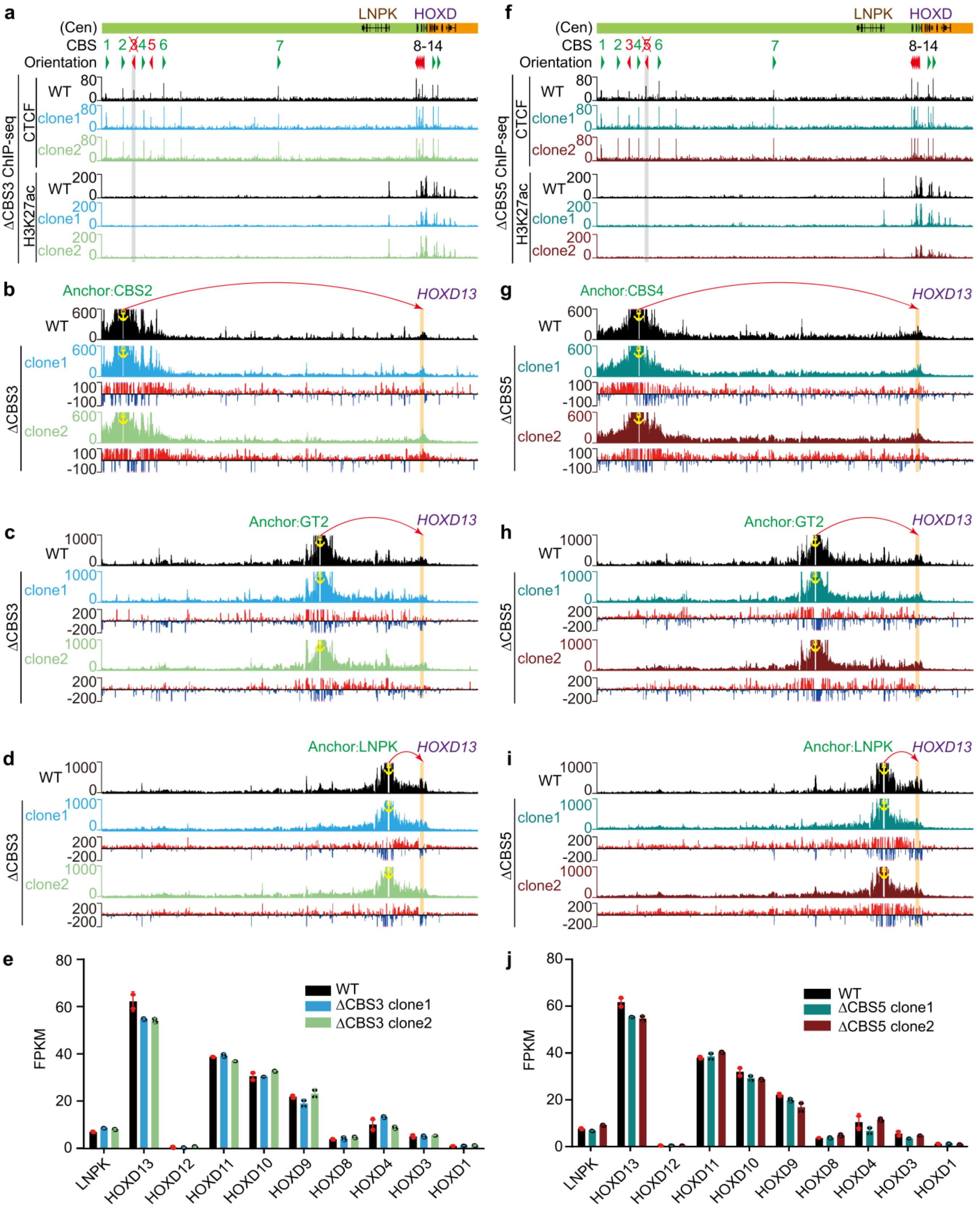

Supplementary Fig. 22. Single knockout of outward-oriented *CBS3* or *CBS5* element downregulates *HOXD13*. a CTCF and H3K27ac ChIP-seq profiles of the *HOXD* centromeric regulatory TAD (C-DOM) in Δ*CBS3* homozygous single-cell clones compared with wild-type (WT) clones. **b**-**d** 4C profiles using *CBS2* (**b**), *GT2* (**c**), or *LNPK* (**d**) element as an anchor in the two Δ*CBS3* single-cell clones compared to WT clones, showing increased chromatin interactions of *HOXD13* with the left boundary and decreased chromatin interactions with the H3K27ac-enriched *GT2 or LNPK* element upon *CBS3* knockout. **e**,**j** Expression levels of *HOXD* genes in Δ*CBS3* (**e**) or Δ*CBS5* (**j**) single-cell clones compared to WT clones. Data as mean ± SD, *$p < 0.05$, **$p < 0.01$, ***$p < 0.001$; two-tailed Student's t test. For each deletion clone, n = 2 biologically independent samples; for their WT controls, n = 4. Source data are provided as a Source Data file. **f** CTCF and H3K27ac ChIP-seq profiles of the C-DOM in Δ*CBS5* single-cell clones compared to WT clones. **g**-**i** 4C profiles using *CBS2* (**g**), *GT2* (**h**), or *LNPK* (**i**) element as an anchor in the two Δ*CBS5* single-cell clones compared to WT clones, showing increased chromatin interactions of *HOXD13* with the left boundary and decreased chromatin interactions with the H3K27ac-enriched *GT2 or LNPK* element upon *CBS5* knockout.

References

1. Bonev, B. *et al.* Multiscale 3D genome rewiring during mouse neural development. *Cell* **171**, 557-572 e24 (2017).

2. Gorkin, D.U. *et al.* An atlas of dynamic chromatin landscapes in mouse fetal development. *Nature* **583**, 744-751 (2020).

3. Zhou, Y., Xu, S., Zhang, M. & Wu, Q. Systematic functional characterization of antisense eRNA of protocadherin alpha composite enhancer. *Genes Dev* **35**, 1383-1394 (2021).
