# Supplementary material for: Outward-oriented sites within clustered CTCF boundaries are key for intra-TAD chromatin interactions and gene regulation
Source: Nat Commun. 2023 Dec 7;14:8101. doi: 10.1038/s41467-023-43849-0 (PMC10703910; doi:10.1038/s41467-023-43849-0)
Supplement: Supplementary file 3 — Description of Additional Supplementary Files [file 41467_2023_43849_MOESM3_ESM.pdf]

Description of Additional Supplementary Files

File Name: Supplementary Data 1

Description: Human pathogenic CNVs covering CBSf or CBS3/CBS5 from ClinGen and DECIPHER databases.

File Name: Supplementary Data 2

Description: Genome-wide CBS orientation patterns of TAD boundaries in human HEC-1-B cells.

File Name: Supplementary Data 3

Description: CBS patterns of TAD boundaries identified in human HEC-1-B cells.

File Name: Supplementary Data 4

Description: Numbers of screened single-cell clones for deletion of outward-oriented CBS elements of HOXD by CRISPR editing.

File Name: Supplementary Data 5

Description: Oligonucleotides used in this study.

File Name: Supplementary Data 6

Description: Antibodies used in this study.
