# Supplementary material for: Outward-oriented sites within clustered CTCF boundaries are key for intra-TAD chromatin interactions and gene regulation
Source: Nat Commun. 2023 Dec 7;14:8101. doi: 10.1038/s41467-023-43849-0 (PMC10703910; doi:10.1038/s41467-023-43849-0)
Supplement: Supplementary file 10 — Reporting Summary [file 41467_2023_43849_MOESM10_ESM.pdf]

nature portfolio

Corresponding author(s): Qiang Wu

Last updated by author(s): Aug 26, 2023

Reporting Summary

Nature Portfolio wishes to improve the reproducibility of the work that we publish. This form provides structure for consistency and transparency in reporting. For further information on Nature Portfolio policies, see our Editorial Policies and the Editorial Policy Checklist.

Statistics

For all statistical analyses, confirm that the following items are present in the figure legend, table legend, main text, or Methods section.

n/a	Confirmed	
☐	☒	The exact sample size (*n*) for each experimental group/condition, given as a discrete number and unit of measurement
☐	☒	A statement on whether measurements were taken from distinct samples or whether the same sample was measured repeatedly
☐	☒	The statistical test(s) used AND whether they are one- or two-sided<br>*Only common tests should be described solely by name; describe more complex techniques in the Methods section.*
☒	☐	A description of all covariates tested
☒	☐	A description of any assumptions or corrections, such as tests of normality and adjustment for multiple comparisons
☐	☒	A full description of the statistical parameters including central tendency (e.g. means) or other basic estimates (e.g. regression coefficient) AND variation (e.g. standard deviation) or associated estimates of uncertainty (e.g. confidence intervals)
☐	☒	For null hypothesis testing, the test statistic (e.g. *F*, *t*, *r*) with confidence intervals, effect sizes, degrees of freedom and *P* value noted<br>*Give P values as exact values whenever suitable.*
☒	☐	For Bayesian analysis, information on the choice of priors and Markov chain Monte Carlo settings
☒	☐	For hierarchical and complex designs, identification of the appropriate level for tests and full reporting of outcomes
☐	☒	Estimates of effect sizes (e.g. Cohen's *d*, Pearson's *r*), indicating how they were calculated

Our web collection on statistics for biologists contains articles on many of the points above.

Software and code

Policy information about availability of computer code

Data collection	No software was used for data collection
Data analysis	For 4C data, Duplicated paired-end reads were removed by FastUniq (version 1.1) program, and only the unique reads were used for analyses using the Bowtie and r3Cseq program. Paired-end reads were aligned to the mouse genome (M. musculus, UCSC mm9) or the human genome (H. sapiens, UCSC hg19) using Bowtie2(version 2.3.5). The sam files were then transformed into bam file using samtools (version 1.15.1).The reads per million (RPM) value was calculated using the r3Cseq program (version 1.20) in the R package (version 3.3.3).<br>For ChIP-seq and ATAC-seq data, Reads were aligned to the mouse genome (M. musculus, UCSC mm9) or the human genome (H. sapiens, UCSC hg19) with modification in the CRISPR editing area if necessary using Bowtie2(version 2.3.5).<br>Sam files were then converted into Bam files using samtools (version 1.15.1) and indexed. BedGraph files were generated using bigWigToBedGraph software with Bam file as input. Bw files were generated using macs14 (version 1.4) software with Bam files as input.<br>For RNA-seq data, Reads were aligned using Hisat2 (version 2.0.4) to the human genome (GRCh38/hg38), The sam files were then transformed into bam file using samtools (version 1.15.1) and the FPKM value was calculated using the Cufflinks program (version2.1.1).<br>Hi-C reads were pre-processed with HiC-Pro (version 3.0.0).

For manuscripts utilizing custom algorithms or software that are central to the research but not yet described in published literature, software must be made available to editors and reviewers. We strongly encourage code deposition in a community repository (e.g. GitHub). See the Nature Portfolio guidelines for submitting code & software for further information.

Data

Policy information about availability of data

All manuscripts must include a data availability statement. This statement should provide the following information, where applicable:

- Accession codes, unique identifiers, or web links for publicly available datasets
- A description of any restrictions on data availability
- For clinical datasets or third party data, please ensure that the statement adheres to our policy

The raw high-throughput sequencing data and processed data generated in this study have been deposited in the GEO database under accession code GSE210817 [https://www.ncbi.nlm.nih.gov/geo/query/acc.cgi?acc=GSE210817]. Source data are provided with this paper. This data has already been publicly released.

Research involving human participants, their data, or biological material

Policy information about studies with human participants or human data. See also policy information about sex, gender (identity/presentation), and sexual orientation and race, ethnicity and racism.

Reporting on sex and gender	No human experiments.
Reporting on race, ethnicity, or other socially relevant groupings	No human experiments.
Population characteristics	No human experiments.
Recruitment	No human experiments.
Ethics oversight	No human experiments.

Note that full information on the approval of the study protocol must also be provided in the manuscript.

Field-specific reporting

Please select the one below that is the best fit for your research. If you are not sure, read the appropriate sections before making your selection.

☒ Life sciences ☐ Behavioural & social sciences ☐ Ecological, evolutionary & environmental sciences

For a reference copy of the document with all sections, see nature.com/documents/nr-reporting-summary-flat.pdf

Life sciences study design

All studies must disclose on these points even when the disclosure is negative.

Sample size	No sample size calculation were performed. We and others normally use 2-3 samples for each experiment.
Data exclusions	No data exclusion.
Replication	For ChIP-seq, ATAC-seq and 4C experiments, two replicates were performed for each sample. For RNA-seq experiments, two or more replicates were performed for each sample. We confirm that all attempts at replications were successful.
Randomization	For all experiments, the samples were allocated into experimental groups randomly.
Blinding	No differences were made when dealing with wildtype and mutant mouse lines or cell lines.

Reporting for specific materials, systems and methods

We require information from authors about some types of materials, experimental systems and methods used in many studies. Here, indicate whether each material, system or method listed is relevant to your study. If you are not sure if a list item applies to your research, read the appropriate section before selecting a response.

Materials & experimental systems

n/a	Involved in the study
☐	☒ Antibodies
☐	☒ Eukaryotic cell lines
☒	☐ Palaeontology and archaeology
☐	☒ Animals and other organisms
☒	☐ Clinical data
☒	☐ Dual use research of concern
☒	☐ Plants

Methods

n/a	Involved in the study
☐	☒ ChIP-seq
☒	☐ Flow cytometry
☒	☐ MRI-based neuroimaging

Antibodies

Antibodies used

CTCF Millipore 07-729 For each experiment,3 ul of antibodies were added with no dilution.
Rad21 Abcam ab992 For each experiment,2 ul of antibodies were added with no dilution.
H3K27ac Abcam ab4729 For each experiment,3 ul of antibodies were added with no dilution.
H3K4me1 Abcam ab8895 For each experiment,3 ul of antibodies were added with no dilution.
H3K4me3 Millipore 17-614 For each experiment,3 ul of antibodies were added with no dilution.
H3K9ac Millipore 06-942 For each experiment,3 ul of antibodies were added with no dilution.
H3K36me3 Abcam ab9050 For each experiment,3 ul of antibodies were added with no dilution.
H3K9me2 Abcam ab1220 For each experiment,3 ul of antibodies were added with no dilution.
H3K9me3 Millipore 07-442 For each experiment,3 ul of antibodies were added with no dilution.

Validation

For CTCF antibody, the company is Millipore with cat. Number 07-729. It is a polyclonal antibody. According to the Millipore website, They routinely evaluate the antibody by Western Blot (Western Blot Analysis: A 1:1000–1:5000 dilution of this lot detected CTCF in HeLa nuclear extract. A previous lot detected CTCF in K562 nuclear extract). For each ChIP-seq experiment, 3 ul of antibodies were added with no dilutions.
For Rad21 antibody, the company is Abcam with cat. Number ab992. It is a polyclonal antibody. According to the Abcam website, they guarantee the antibody has been tested for IP and WB. For each ChIP-seq experiment, 2 ul of antibodies were added with no dilutions.
For H3K27ac antibody, the company is Abcam with cat. Number ab4729. It is a polyclonal antibody. According to the Abcam website, they guarantee the antibody has been tested for ICC/IF, WB, IHC-P, ChIP and Preparr. For each ChIP-seq experiment, 3 ul of antibodies were added with no dilutions.
For H3K4me1 antibody, the company is Abcam with cat. Number ab8895. It is a polyclonal antibody. According to the Abcam website, they guarantee the antibody has been tested for ICC/IF, ChIP, WB and IHC-P. For each ChIP-seq experiment, 3 ul of antibodies were added with no dilutions.
For H3K4me3 antibody, the company is Millipore with cat. Number 17-614. It is a monoclonal antibody (clone name unprovided according to Merck). According to the Millipore website, they routinely evaluate the antibody by Chromatin Immunoprecipitation (Sonicated chromatin prepared from untreated HeLa cells (1 X 106 cell equivalents) was subjected to chromatin immunoprecipitation using 3 µL of either a normal rabbit IgG or 3 µL Anti-Trimethyl-Histone H3 (Lys4) Monoclonal IgG and the Magna ChIP A (Part # 17-610) Kit. Successful immunoprecipitation of trimethyl-histone H3 (Lys4) associated DNA fragments was verified by qPCR using control ChIP Primers flanking the human GAPDH promoter). For each ChIP-seq experiment, 3 ul of antibodies were added with no dilutions.
For H3K9ac antibody, the company is Millipore with cat. Number 06-942. It is a polyclonal antibody. According to the Millipore website, they routinely evaluate the antibody by Western Blotting Analysis (A 1:10,000 dilution of this antibody detected acetyl-Histone H3 (Lys9) in 10 µg of sodium butyrate treated HeLa cell lysate). For each ChIP-seq experiment, 3 ul of antibodies were added with no dilutions.
For H3K36me3 antibody, the company is Abcam with cat. Number ab9050. It is a polyclonal antibody. According to the Abcam website, they guarantee the antibody has been tested for ICC/IF, WB and ChIP. For each ChIP-seq experiment, 3 ul of antibodies were added with no dilutions.
For H3K9me2 antibody, the company is Abcam with cat. Number ab1220. It is a monoclonal antibody with clone number mAbcam 1220. According to the Abcam website, they guarantee the antibody has been tested for ICC/IF, WB, ELISA, IHC-P and ChIP. For each ChIP-seq experiment, 3 ul of antibodies were added with no dilutions.
For H3K9me3 antibody, the company is Millipore with cat. Number 07-442. It is a polyclonal antibody. According to the Millipore website, they routinely evaluate the antibody by Western Blot on HeLa acid extract and recombinant Histone H3 (cat. # 14-411) (Western Blot Analysis:
1:500 dilution of this lot detected trimethyl Histone H3 on 10 µg of HeLa acid extract but not on recombinant Histone H3). For each ChIP-seq experiment, 3 ul of antibodies were added with no dilutions.

Eukaryotic cell lines

Policy information about cell lines and Sex and Gender in Research

Cell line source(s): HEK293T

Authentication: None

Mycoplasma contamination	Not tested.
Commonly misidentified lines (See ICLAC register)	None

Animals and other research organisms

Policy information about studies involving animals; ARRIVE guidelines recommended for reporting animal research, and Sex and Gender in Research

Laboratory animals	C57BL/6J ICR<br>Mice were maintained in an SPF mouse facility at 23 °C, with a humidity between 40 and 60 %, with a 12 h (7:00-19:00) light /12 h (19:00-7:00) dark cycle. 6-week-old C57BL/6J female mice were super-ovulated and mated with the sterilized C57BL/6J male mice to produce enough embryos. The F0 mice of 2-month old with desired deletions were mated with wildtype C57BL/6J mice to generate heterozygous F1 mice. For expression mapping at different embryonic states during cortical development, E12.5-P21.5 mice were used, for other studies P0.5 mice were used.
Wild animals	None
Reporting on sex	We carried out experiments and found no significant difference between male and female mice in expression of cPcdh genes. Gender is not concerned in following experiments.
Field-collected samples	No field collected samples were used in the study.
Ethics oversight	All the mouse experiments were approved by the Institutional Animal Care and Use Committee (IACUC) of Shanghai Jiao Tong University (Protocol#: 1602029).

Note that full information on the approval of the study protocol must also be provided in the manuscript.

Plants

Seed stocks	No plant experiments
Novel plant genotypes	No plant experiments
Authentication	No plant experiments

ChIP-seq

Data deposition

☒ Confirm that both raw and final processed data have been deposited in a public database such as GEO.

☒ Confirm that you have deposited or provided access to graph files (e.g. BED files) for the called peaks.

Data access links<br>*May remain private before publication.*	Raw data and processed data could be downloaded at https://www.ncbi.nlm.nih.gov/geo/query/acc.cgi?acc=GSE210817 .

Files in database submission

GSM6438598	ChIP_F_DEL_H3K36me3_rep1
GSM6438600	ChIP_F_DEL_H3K4me1_rep1
GSM6438602	ChIP_F_DEL_H3K4me3_rep1
GSM6438604	ChIP_F_DEL_H3K9ac_rep1
GSM6438605	ChIP_F_DEL_H3K9ac_rep2
GSM6438608	ChIP_F_DEL_H3K9me2_rep1
GSM6438610	ChIP_F_DEL_H3K9me3_rep1
GSM6438616	ChIP_FG_DEL_H3K36me3_rep1
GSM6438617	ChIP_FG_DEL_H3K36me3_rep2
GSM6438618	ChIP_FG_DEL_H3K4me1_rep1
GSM6438620	ChIP_FG_DEL_H3K4me3_rep1
GSM6438621	ChIP_FG_DEL_H3K4me3_rep2
GSM6438622	ChIP_FG_DEL_H3K9ac_rep1
GSM6438626	ChIP_FG_DEL_H3K9me2_rep1
GSM6438628	ChIP_FG_DEL_H3K9me3_rep1
GSM6438634	ChIP_WT_H3K36me3_rep1
GSM6438636	ChIP_WT_H3K4me1_rep1
GSM6438638	ChIP_WT_H3K4me3_rep1
GSM6438639	ChIP_WT_H3K4me3_rep2
GSM6438640	ChIP_WT_H3K9ac_rep1
GSM6438641	ChIP_WT_H3K9ac_rep2
GSM6438644	ChIP_WT_H3K9me2_rep1
GSM6438645	ChIP_WT_H3K9me2_rep2
GSM6438646	ChIP_WT_H3K9me3_rep1

GSM6438647	ChIP_WT_H3K9me3_rep2
GSM6438648	ChIP_WT_Rad21_rep1
GSM6438650	ChIP_f_del_K27ac_rep1
GSM6438651	ChIP_f_del_K27ac_rep2
GSM6438652	ChIP_WT_for_f_and_fg_del_K27ac_rep1
GSM6438653	ChIP_WT_for_f_and_fg_del_K27ac_rep2
GSM6438654	ChIP_FG_del_K27ac_rep1
GSM6438655	ChIP_FG_del_K27ac_rep2
GSM6438656	ChIP_A_K27ac_rep1
GSM6438657	ChIP_A_K27ac_rep2
GSM6438658	ChIP_BC_K27ac_rep1
GSM6438659	ChIP_BC_K27ac_rep2
GSM6438660	ChIP_D_K27ac_rep1
GSM6438661	ChIP_D_K27ac_rep2
GSM6438662	ChIP_E_K27ac_rep1
GSM6438663	ChIP_E_K27ac_rep2
GSM6438664	ChIP_BE_K27ac_rep1
GSM6438665	ChIP_BE_K27ac_rep2
GSM6438666	ChIP_DE_K27ac_rep1
GSM6438667	ChIP_DE_K27ac_rep2
GSM6438668	ChIP_BCD_K27ac_rep1
GSM6438669	ChIP_BCD_K27ac_rep2
GSM6438670	ChIP_WT_K27ac_rep1
GSM6438671	ChIP_WT_K27ac_rep2
GSM6438672	ChIP_HS7L_K27ac_rep1
GSM6438673	ChIP_HS7L_K27ac_rep2
GSM6438674	ChIP_WT_CTCF_rep1
GSM6438675	ChIP_WT_CTCF_rep2
GSM6438676	ChIP_WT_CTCF_rep3
GSM6438677	ChIP_WT_CTCF_rep4
GSM6438678	ChIP_WT_CTCF_rep5
GSM6438679	ChIP_WT_CTCF_rep6
GSM6438680	ChIP_WT_CTCF_rep7
GSM6438681	ChIP_WT_CTCF_rep8
GSM6438682	ChIP_A_CTCF_rep1
GSM6438683	ChIP_A_CTCF_rep2
GSM6438684	ChIP_F_CTCF_rep1
GSM6438685	ChIP_F_CTCF_rep2
GSM6438686	ChIP_FG_CTCF_rep1
GSM6438687	ChIP_FG_CTCF_rep2
GSM7042582	ChIP_CBSa_CTCF_rep1
GSM7042583	ChIP_CBSa_CTCF_rep2
GSM7042584	ChIP_CBSa_K27ac_rep1
GSM7042585	ChIP_CBSa_K27ac_rep2
GSM7042586	ChIP_F_del_CTCF_rep1
GSM7042587	ChIP_F_del_CTCF_rep2
GSM7042588	ChIP_FG_del_CTCF_rep1
GSM7042589	ChIP_FG_del_CTCF_rep2
GSM7042590	ChIP_HS18_del_K27ac_rep1
GSM7042591	ChIP_HS18_del_K27ac_rep2
GSM7042592	ChIP_HS18_to_HS20_del_K27ac_rep1
GSM7042593	ChIP_HS18_to_HS20_del_K27ac_rep2
GSM7042594	ChIP_HS19_to_HS20_del_K27ac_rep1
GSM7042595	ChIP_HS19_to_HS20_del_K27ac_rep2
GSM7042596	ChIP_HS51bL_del_K27ac_rep1
GSM7042597	ChIP_HS51bL_del_K27ac_rep2
GSM7042598	ChIP_HS51bL_to_HS18_del_K27ac_rep1
GSM7042599	ChIP_HS51bL_to_HS18_del_K27ac_rep2
GSM7042600	ChIP_HS51bL_to_HS20_del_K27ac_rep1
GSM7042601	ChIP_HS51bL_to_HS20_del_K27ac_rep2
GSM7042602	ChIP_HS7L_del_K27ac_rep1
GSM7042603	ChIP_HS7L_del_K27ac_rep2
GSM7042604	ChIP_WT_CTCF_rep10
GSM7042605	ChIP_WT_CTCF_rep11
GSM7042606	ChIP_WT_CTCF_rep9
GSM7042607	ChIP_WT_K27ac_rep3
GSM7042608	ChIP_WT_K27ac_rep4
GSM7042609	ChIP_WT_K27ac_rep5
GSM7042610	ChIP_WT_K27ac_rep6
GSM7042611	ChIP_WT_K27ac_rep7
GSM7042612	ChIP_WT_K27ac_rep8
GSM7042614	ChIP_F_DEL_H3K36me3_rep2
GSM7042615	ChIP_F_DEL_H3K4me1_rep2
GSM7042616	ChIP_F_DEL_H3K4me3_rep2
GSM7042619	ChIP_F_DEL_H3K9me2_rep2
GSM7042620	ChIP_F_DEL_H3K9me3_rep2

GSM7042624	ChIP_FG_DEL_H3K4me1_rep2
GSM7042626	ChIP_FG_DEL_H3K9ac_rep2
GSM7042628	ChIP_FG_DEL_H3K9me2_rep2
GSM7042629	ChIP_FG_DEL_H3K9me3_rep2
GSM7042632	ChIP_WT_H3K36me3_rep2
GSM7042633	ChIP_WT_H3K4me1_rep2
GSM7042634	ChIP_WT_H3K4me3_rep2
GSM7042635	ChIP_WT_H3K9ac_rep2
GSM7042637	ChIP_WT_H3K9me2_rep2
GSM7042638	ChIP_WT_H3K9me3_rep2
GSM7042639	ChIP_WT_Rad21_rep2
GSM7730728	293T_WT_CTCF_rep1
GSM7730729	293T_WT_CTCF_rep2
GSM7730732	293T_WT_H3K27ac_rep1
GSM7730733	CTCF_CBS3_del_rep1
GSM7730736	CTCF_CBS5_del_rep2
GSM7730737	CTCF_del_sample1_rep1
GSM7730738	CTCF_del_sample1_rep2
GSM7730739	CTCF_del_sample2_rep1
GSM7730740	CTCF_del_sample2_rep2
GSM7730741	CTCF_del_sample3_rep1
GSM7730742	CTCF_del_sample3_rep2
GSM7730743	H3K27ac_293T_WT_for_double_CBS_KO_rep1
GSM7730744	H3K27ac_293T_WT_for_double_CBS_KO_rep2
GSM7730745	H3K27ac_293T_WT_for_single_CBS_KO_rep1
GSM7730748	H3K27ac_CBS3_KO_alone_sample1
GSM7730750	H3K27ac_CBS5_KO_alone_sample1
GSM7730751	H3K27ac_double_KO_del_sample1_rep1
GSM7730752	H3K27ac_double_KO_del_sample1_rep2
GSM7730753	H3K27ac_double_KO_del_sample2_rep1
GSM7730754	H3K27ac_double_KO_del_sample2_rep2
GSM7730755	H3K27ac_double_KO_del_sample3_rep1
GSM7730756	H3K27ac_double_KO_del_sample3_rep2
GSM7759584	ChIP_CBSF_del_Rad21_rep1
GSM7759585	ChIP_CBSF_del_Rad21_rep2
GSM7759587	ChIP_FG_both_DEL_Rad21_rep1
GSM7759589	ChIP_FG_both_DEL_Rad21_rep2
GSM7759590	ChIP_g_del_CTCF_rep1
GSM7759591	ChIP_g_del_CTCF_rep2
GSM7759592	ChIP_g_del_Rad21_rep1
GSM7759593	ChIP_g_del_Rad21_rep2
GSM7759594	ChIP_g_del_Rad21_rep3
GSM7759595	ChIP_g_del_Rad21_rep4
GSM7759596	ChIP_WT_for_g_del_CTCF_rep1
GSM7759597	ChIP_WT_for_g_del_CTCF_rep2
GSM7759598	ChIP_WT_for_g_del_Rad21_rep1
GSM7759599	ChIP_WT_for_g_del_Rad21_rep2

Genome browser session
(e.g. UCSC)

N/A

Methodology

Replicates

2

Sequencing depth

2*150 bp

Antibodies

CTCF Millipore 07-729
Rad21 Abcam ab992
H3K27ac Abcam ab4729
H3K4me1 Abcam ab8895
H3K4me3 Millipore 17-614
H3K9ac Millipore 06-942
H3K36me3 Abcam ab9050
H3K9me2 Abcam ab1220
H3K9me3 Millipore 07-442

Peak calling parameters

bowtie -p 24 -m 2 -n 1 -chunkmbs 10000 -q reference file input file samfiles
samtools sort -@ 24 -o output file samfile
macs14 -t bamfiles -f BAM -g hs/mm -o output file -B -w
samtools index
bamCoverage -p 24 -skipNAs --binsize 20 --smoothlength 60

Data quality	N/A
Software	bowtie (version 1.2.3) macs14
